# MYSM1 Suppresses Cellular Senescence and the Aging Process to Prolong Lifespan

**DOI:** 10.1002/advs.202001950

**Published:** 2020-09-30

**Authors:** Mingfu Tian, Yuqing Huang, Yunting Song, Wen Li, Peiyi Zhao, Weiyong Liu, Kailang Wu, Jianguo Wu

**Affiliations:** ^1^ State Key Laboratory of Virology College of Life Sciences Wuhan University Wuhan 430072 China; ^2^ Guangdong Provincial Key Laboratory of Virology Institute of Medical Microbiology Jinan University Guangzhou 510632 China

**Keywords:** aging, DNA repair, Myb‐like, SWIRM, and MPN domains‐containing protein 1 (MYSM1), senescence, senescence‐associated secretory phenotype (SASP)

## Abstract

Aging is a universal feature of life that is a major focus of scientific research and a risk factor in many diseases. A comprehensive understanding of the cellular and molecular mechanisms of aging are critical to the prevention of diseases associated with the aging process. Here, it is shown that MYSM1 is a key suppressor of aging and aging‐related pathologies. MYSM1 functionally represses cellular senescence and the aging process in human and mice primary cells and in mice organs. MYSM1 mechanistically attenuates the aging process by promoting DNA repair processes. Remarkably, MYSM1 deficiency facilitates the aging process and reduces lifespan, whereas MYSM1 over‐expression attenuates the aging process and increases lifespan in mice. The functional role of MYSM1 is demonstrated in suppressing the aging process and prolonging lifespan. MYSM1 is a key suppressor of aging and may act as a potential agent for the prevention of aging and aging‐associated diseases.

## Graphical abstract

Aging is a universal feature of life and a risk factor for many diseases. This study reveals that MYSM1 plays a key role in the suppression of the aging process and in the prolongation of lifespan. Thus, MYSM1 may act as a potential agent for the prevention of the aging process and the treatment of age‐associated diseases.

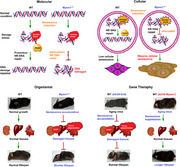

## 1 Introduction

Aging is characterized by a functional decline across multiple organ systems and is a risk factor for many human diseases.^[^ ^1^, ^2^ ^]^ Substantial evidence has demonstrated that senescence is a key hallmark of the aging process and plays a critical roles in controlling aging and aging‐associated diseases.^[^ ^3^, ^4^, ^5^, ^6^, ^7^ ^]^ Senescence is a cellular response that acts to restrict the proliferation of aged and damaged cells,^[^ ^8^, ^9^ ^]^ and is also a state of growth arrest and pro‐inflammatory cytokine release in response to stresses.^[^ ^10^ ^]^ One hallmark of cellular senescence is the secretion of excessive proinflammatory cytokines, chemokines, extracellular matrix proteins, growth factors, and proteases termed the senescence‐associated secretory phenotype (SASP).^[^ ^11^ ^]^ Senescence constitutes a stress response triggered by insults associated with aging including genomic instability and telomere attrition.^[^ ^12^ ^]^

DNA damage is a causal factor in the aging process that drives cells into senescence or apoptosis as results of the DNA damage response (DDR) controlled by DNA repair processes.^[^ ^13^ ^]^ DNA double‐strand break (DSB) repair is known to decline age,^[^ ^14^ ^]^ leading to the accumulation of genomic rearrangements.^[^ ^15^ ^]^ Mutations in DNA DSB repair genes reduce lifespan, indicating that DNA repair pathways play a critical roles in the aging process.^[^ ^16^ ^]^

The Myb‐like, SWIRM, and MPN domains‐containing protein 1 (MYSM1) is a histone 2A (H2A) deubiquitinase that specifically deubiquitinates K119‐monoubiquitinated H2A.^[^ ^17^, ^18^, ^19^, ^20^ ^]^ It is a key functional regulator of hematopoietic stem cells, lymphocytes, and blood cells, and serves as an important regulator of tissue differentiation.^[^ ^21^, ^22^ ^]^ MYSM1 is also linked to heritable bone marrow failure syndromes,^[^ ^23^, ^24^, ^25^ ^]^ plays a role in regulating skin development in mice,^[^ ^26^ ^]^ and impedes antiviral signaling.^[^ ^17^ ^]^ Loss of Mysm1 has been shown to promote activation of the p53 stress response and induced abnormal cell development and tissue differentiation.^[^ ^27^, ^28^, ^29^, ^30^ ^]^ More recently, a study revealed that Mysm1 levels increase in response to etoposide‐induced DNA damage and that mice lacking Mysm1 show a shorter lifespan.^[^ ^31^ ^]^ These important roles of MYSM1 implicate that it may be involved in the regulation of cellular senescence and the aging process.

The present study showed that MYSM1 is a key suppressor of senescence and aging. Functionally, MYSM1 functionally represses DDR‐associated SASP and the aging process. Mechanistically, MYSM1 represses the aging process by promoting homologous recombination (HR) mediated DNA repair. Mysm1 deficiency promotes aging and aging‐related pathologies and reduces lifespan in mice. AAV9‐Mysm1 was shown to attenuate the aging process to prolong the lifespan of mice. Our data suggest that Mysm1 is a potential agent for the prevention of aging and aging‐related diseases.

## 2 Results

### 2.1 MYSM1 Promotes Mediated HR DNA Repair

**Figure 1:**
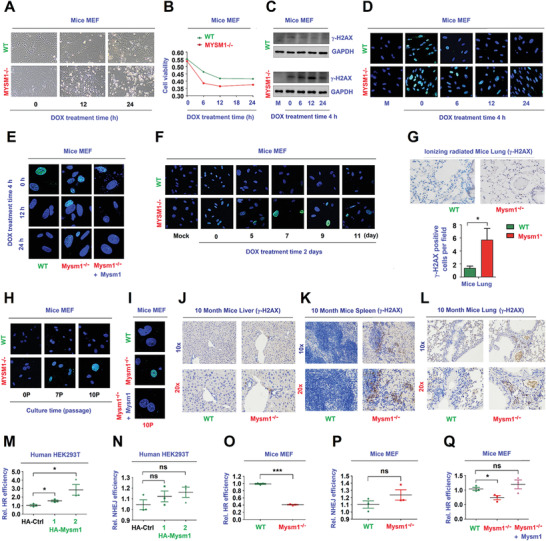
MYSM1 promotes HR mediated DNA repair. A,B) WT MEFs or Mysm1^−/−^ MEFs were treated with DOX. (A) Cell images showing the cell cytotoxicity. Cell viability was measured using the CELL COUNTING kit‐8 B). C–E) WT MEFs and Mysm1^−/−^ MEFs (C,D) or WT MEFs, Mysm1^−/−^ MEFs, and Mysm1^−/−^ MEFs transfected with Flag‐Mysm1 (E) were treated with DOX to induce DNA damage. *γ*‐H2AX and GAPDH protein levels were detected by Western blotting (WB) (C). *γ*‐H2AX protein was detected by anti‐*γ*‐H2AX antibody and visualized under a confocal microscope (D,E). F) MEFs were treated with DOX for 2 days to induce DNA damage. *γ*‐H2AX protein was detected by anti‐*γ*‐H2AX antibody and visualized under confocal microscope. G) 2 month old WT and Mysm1^−/−^ mice (*n* = 3 per group) were subjected to IR. The levels of *γ*‐H2AX in the lung were determined by IHC. Scale bars, 50 µm. H) MEFs were grown continuously for different passages to induce DNA damage. *γ*‐H2AX levels were detected and visualized under a confocal microscope. I) MEFs were grown continuously for 10 passages to induce DNA damage. *γ*‐H2AX levels were detected and visualized under a confocal microscope. J,K) Liver, spleen, and lung were excised from 10 month old WT and Mysm1^−/−^ mice (*n* = 3 per group). The levels of *γ*‐H2AX were determined by IHC. Scale bars, 100 × = 100 µm, 200 × = 50 µm. M–P) Human HEK293T cells (M,N) and mice MEFs (O,P) were co‐transfected with a HR reporter system (M,O) or a NHEJ reporter system (N,P), along with pCMV‐DsRed and HA‐Ctrl or HA‐Mysm1. The efficiencies of HR (M) and NHEJ (N) were determined. Q) MEFs were co‐transfected with the HR reporter along with pCMV‐DsRed and Flag‐Ctrl or Flag‐Mysm1. The efficiencies of HR were determined. Data are presented as the means ±SD. Statistical analyses were performed using Prism software by Unpaired *t*‐tests. *p < 0.05, **p < 0.01, ***p < 0.001, n.s. = no significance.

DNA damage is a causal factor of the aging process, and we initially explored the link between Mysm1 expression and the DNA damage response using a Mysm1‐knockout (KO) mouse model (Mysm1^−/−^ C57BL/6).^[^ ^30^ ^]^ Murine embryonic fibroblasts (MEFs) isolated from Mysm1^−/−^ and WT (C57BL/6) mice were treated with etoposide (ETO) and Doxorubicin (DOX), which induce senescence.^[^ ^32^ ^]^ We showed that cell cytotoxicity increased (**Figure** **1**A) and cell viability decreased (Figure 1B) in Mysm1^−/−^ MEFs as compared to WT MEFs, indicating that Mysm1 deficient MEFs are more sensitive to DNA damage.

Previously, it has been reported that DNA repair declines with age and plays a central role in senescence and the aging process,^[^ ^13^ ^]^ and SIRT6 has been shown to regulate DNA repair in male mice with SIRT6 deficiency resulting in genomic instability and an aging‐like phenotype.^[^ ^33^, ^34^, ^35^ ^]^ However, the molecular traits involved in the suppression of aging remain largely unknown.

Next, we determined the role of MYSM1 in the regulation of DNA repair. The *γ*‐H2AX protein, a marker of DNA damage,^[^ ^33^ ^]^ was induced initially by DOX treatment, and then reduced at 24 h post‐treatment in WT MEFs. In contrast, *γ*‐H2AX remained at a higher level from 6 to 24 h post‐treatment in Mysm1^−/−^ MEFs (Figure 1C,D and Figure S1A, Supporting Information). Interestingly, it was observed that like in WT MEFs, DOX‐induced *γ*‐H2AX was repressed at 24 h post‐treatment in Mysm1^−/−^ MEFs transfected with Mysm1 (Figure 1E and Figure S1B,C, Supporting Information). These results suggested that MYSM1 plays a role in attenuating the DNA damage response and promoting DNA repair. DOX‐induced *γ*‐H2AX was reduced in WT MEFs from 5 to 11 days post‐treatment, whereas it remained higher level in Mysm1^−/−^ MEFs from 5 to 11 days post‐treatment (Figure 1F and Figure S1D, Supporting Information), indicating that Mysm1 deficiency fails to protect cells from DNA damage. Notably, *γ*‐H2AX levels were higher in Mysm1^−/−^ mice lung tissues compared to WT mice lung following exposure to ionizing radiation (IR), a known stimulus of senescence,^[^ ^32^ ^]^ (Figure 1G). Moreover, *γ*‐H2AX remained at a higher level in Mysm1^−/−^ MEFs relative to WT MEFs after culturing for 7 and 10 passages (Figure 1H and Figure S1E, Supporting Information). Similar to the response in WT MEFs, *γ*‐H2AX was attenuated in Mysm1^−/−^ MEFs transfected with Mysm1 at 10P (Figure 1I and Figure S1F,G, Supporting Information). Moreover, *γ*‐H2AX levels were higher in the liver, spleen, and lung of 10‐month‐ old Mysm1^−/−^ mice relative to WT mice tissues (Figure 1J–L and Figure S1H, Supporting Information). Collectively, these results revealed that MYSM1 deficiency represses DNA repair, whereas MYSM1 over‐expression promotes DNA repair.

DNA repair is mediated by two major molecular pathways: homologous recombination (HR), and non‐homologous end joining (NHEJ).^[^ ^36^ ^]^ Here, the effects of MYSM1 on the regulation of DNA repair pathways were evaluated using the HR reporter system and the NHEJ reporter system.^[^ ^33^, ^37^ ^]^ In transfected HEK293T cells, Mysm1 promoted HR efficiency (Figure 1M), but it had no effect on the efficiency of NHEJ (Figure 1N). In Mysm1^−/−^ mice MEFs, HR efficiency was repressed (Figure 1O), whilst NHEJ efficiency remained relatively unchanged (Figure 1P). Importantly, the repression of HR efficiency in Mysm1^−/−^ MEFs was abrogated by transfection of Mysm1 (Figure 1Q). Taken together, the results demonstrate that MYSM1 prevents DNA damage accumulation by facilitating HR mediated DNA repair.

### 2.2 MYSM1 Correlates with Senescence

**Figure 2:**
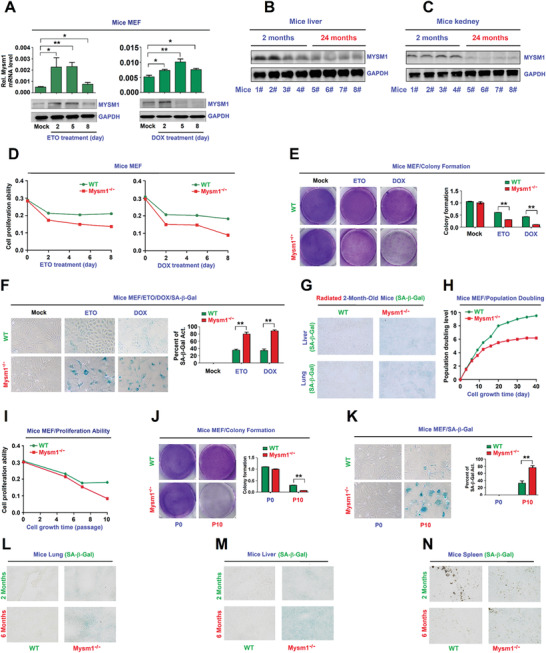
MYSM1 correlates with senescence. A–C) C57BL/6 mice MEFs were treated with ETO or DOX (A). Kidney (B) and lung (C) tissues were excised from 2‐ and 22‐month‐old WT mice (*n* = 4 per group). MYSM1 and GAPDH protein levels were assessed by WB. D–F) WT or Mysm1^−/−^ mice MEFs were pre‐treated with ETO or DOX for 48 h to induce senescence. Cell proliferation was determined (D). Colony formation was analyzed by crystal violet staining (E). SA‐*β*‐Gal activity was determined by X‐gal staining (F). G) 2 month old WT and Mysm1^−/−^ mice were exposed to ionizing radiation (*n* = 3 per group). SA‐*β*‐Gal activities in the lung and liver were determined by IHC. H–K) WT or Mysm1^−/−^ MEFs were grown continuously for different passages (P) or on different days (D). Population doubling levels were determined (H). Cell proliferation was measured (I). Colony formation was analyzed (J). SA‐*β*‐Gal activities were determined by X‐gal staining (K). L–N) SA‐*β*‐Gal activities in the lung (L), liver (M), and spleen (N) of 2‐ and 6‐month‐old WT and Mysm1^−/−^ mice were determined by IHC analysis (*n* = 3 per group). Scale bars = 50 µm. Data presented as the mean ± SD. Statistical analyses were performed using an Unpaired *t*‐test in Prism software. *p < 0.05, **p < 0.01, ***p < 0.001. n.s. = no significance.

DNA damage is key aspect of the aging process that drives cells into apoptosis or senescence as outcomes of DNA damage response (DDR),^[^ ^32^, ^34^, ^37^ ^]^ and therefore we explored the relationship between Mysm1 expression and senescence. In human primary diploid fibroblasts (WI‐38), treatments with ETO and DOX increased Mysm1 expression after 2 and 5 days, but decreased Mysm1 expression at 8 days (Figure S2A,B, Supporting Information). Similarly, in WT (C57BL/6) mice MEFs, Mysm1 mRNA, and MYSM1 protein shown to increase after 2 and 5 days, and decreased at 8 days after ETO and DOX treatments (**Figure** **2**A and Figure S2C, Supporting Information). Also, Mysm1 mRNA and MYSM1 protein were notably lower in the kidney and lung of 22 month old mice, compared to mice at 2 months old (Figure 2B,C). p16^Ink4a^ (p16) and p21^Cip1^ (p21) are known as key senescence biomarkers.^[^ ^38^ ^]^ SASP‐associated cytokines including IFN‐1*β*, Cxcl10, IL‐6, IL‐1*α*, IL‐1*β*, and CCL2, were continuously induced in MEFs from 2 to 8 days upon ETO and DOX treatments (Figure S2D,E, Supporting Information). These results suggest that Mysm1 expression correlates with cellular senescence.

The effect of MYSM1 on senescence was explored in Mysm1^−/−^ mice, in which Mysm1 mRNA and MYMS1 protein were hardly detected (Figure S2F, Supporting Information). After the treatment with ETO or DOX, cell proliferation and colony formation were decreased significantly faster in Mysm1^−/−^ MEFs relative to WT MEFs (Figure 2D,E). However, upon treatments, the activities of senescence‐associated *β*‐galactosidase (SA‐*β*‐Gal), a biomarker of cellular senescence,^[^ ^39^ ^]^ were significantly higher in Mysm1^−/−^ MEFs compared to WT cells (Figure 2F) and in human WI‐38 cells stably expressing a short hairpin RNA targeting Mysm1 (sh‐Mysm1) (Figure S2G,H, Supporting Information). Upon exposure to ionizing radiation (IR), SA‐*β*‐gal activities were induced in the liver and lungs of Mysm1^−/‐^ mice, but hardly detected in WT mice tissues (Figure 2G). These findings indicated that MYSM1 promotes cell proliferation and colony formation and attenuates SA‐*β*‐Gal activation to repress senescence.

The biological role of Mysm1 in the aging process was assessed in MEFs at different times or passages. Population doubling levels decreased faster in Mysm1^−/‐^ MEFs compared to WT MEFs from 10 to 40 days (Figure 2H). After culturing for 10 passages (10P), cell proliferation (Figure 2I) and colony formation (Figure 2J) were significantly lower, whilst SA‐*β*‐Gal activities (Figure 2K) were higher in Mysm1^−/−^ MEFs. Also, SA‐*β*‐gal activities were induced the lung, liver and spleen of 6 month old Mysm1^−/‐^ mice, slightly elevated in 2 month old Mysm1^−/‐^ mice, but Mysm1 levels were barely detectable in 2 and 6 month old WT mice tissues (Figure 2L–N). These findings indicated that loss of MYSM1 promotes senescence cell accumulation in mice tissues. Together, the results suggested that Mysm1 expression is associated with senescence, and that MYSM1 prevents DNA damage accumulation to suppress senescence and aging by facilitating the HR mediated DNA repair.

### 2.3 MYSM1 Suppresses DDR‐Associated SASP

**Figure 3:**
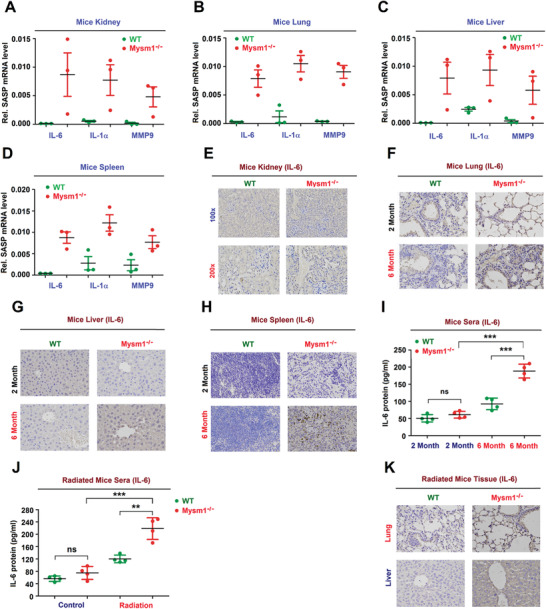
MYSM1 suppresses DDR‐associated SASP. A–D) IL‐6, IL‐1*β*, and MMP3 mRNA levels in the kidney (A), lung (B), liver (C), and spleen (D) excised from 6‐month‐old WT and Mysm1^−/−^ mice (*n* = 3 per group) determined by RT‐PCR. E–H) IL‐6 protein in the E) kidney, F) lung, G) liver, and H) spleen excised from 6‐month‐old WT and Mysm1^−/−^ mice (*n* = 3 per group) analyzed by IHC. Scale bars = 50 µm. I) IL‐6 protein in the sera from 6‐month‐old WT and Mysm1^−/−^ mice (*n* = 3 per group) measured by ELISA. J,K) 2‐month‐old WT and Mysm1^−/−^ mice were exposed to IR (*n* = 3 per group). IL‐6 protein in the sera was measured by ELISA (J). IL‐6 protein in the lung and liver was analyzed by IHC. Scale bars = 50 µm (K). Data are means ± SD. Statistical analyses were performed using an Unpaired *t*‐test in Prism software. *p < 0.05, **p < 0.01, ***p < 0.001. n.s. = no significance.

As DNA damage accumulation is an important factor that induces SASP,^[^ ^40^ ^]^ the functional relevance of endogenous MYSM1 in the regulation of DDR‐associated SASP was evaluated. Upon treatment with DOX or ETO, IL‐1*α*, IL‐6, and MMP3 mRNA levels were notably higher in WI‐38 cells stably expressing sh‐Mysm1 (Figure S3A–C, Supporting Information), and the levels of IFN‐*β*, Cxcl10, IL‐1*α*, IL‐1*β*, and CCL2 mRNAs were significantly higher in Mysm1^−/−^ mice MEFs (Figure S3D–H, Supporting Information). The mRNA levels of the cytokines were notably higher in Mysm1^−/−^ MEFs after culturing for 10 passages (Figure S3I,M, Supporting Information). ETO‐induced p16, p21, IL‐1*α*, and IL‐1*β* mRNAs were repressed in MEFs transfected with Flag‐Mysm1 (**Figure** **3**N–R). These results indicate that Mysm1 knock‐down or knock‐out facilitates SASP, whilst Mysm1 over‐expression attenuates SASP upon induction of senescence and during aging process.

Notably, IL‐6, IL‐1*α*, and MMP3 mRNAs were expressed at higher levels in the kidney, lung, liver, and spleen of Mysm1^−/−^ mice compared to WT tissues (Figure 3A–D). The level of IL‐6 was much higher in 6‐month‐old Mysm1^−/−^ mice tissues compared to WT tissues, whereas no differences in the level of IL‐6 were observed in 2 month old Mysm1^−/−^ and WT tissues (Figure 3E–H). Similarly, the serum levels of IL‐6 protein were significantly higher in both 6 month old Mysm1^−/−^ mice compared to 6 month old WT mice, whereas IL‐6 levels were comparable in both 2 month old Mysm1^−/−^ and WT mice (Figure 3I). Furthermore, IL‐6 protein was highly induced in the serum, lung, and liver of Mysm1^−/−^ mice following exposure to ionizing radiation (Figure 3J,K). Taken together, these results showed that MYSM1 represses DDR‐associated SASP.

### 2.4 MYSM1 Represses Senescence in Mice

**Figure 4:**
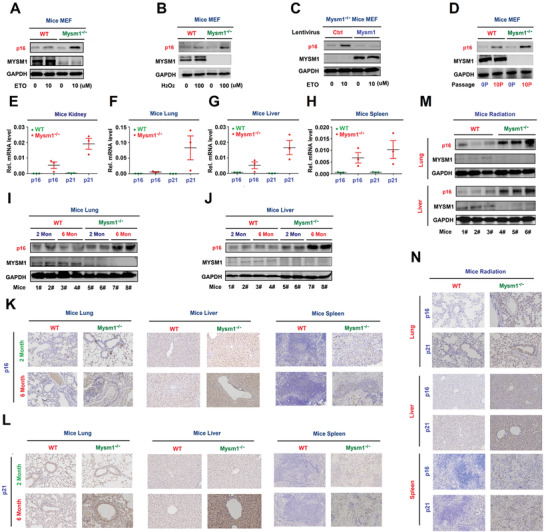
MYSM1 represses senescence in aged mice. A–D) MEFs were treated with ETO (A) or H_2_O_2_ (B), infected with lentivirus expressing Mysm1 (C), or grown for different passages (D). p16, MYSM1, and GAPDH proteins were detected by WB. E–H) p16 and p21 mRNA in the kidney (E), lung (F), liver (G), and spleen (H) of 6 month old WT and Mysm1^−/−^ mice (*n* = 3 per group) were determined by RT‐PCR. I,J) p16, MYSM1, and GAPDH proteins in the lung (I) and liver (J) of 2 and 6 month old WT and Mysm1^−/−^ mice (*n* = 3 per group) were detected by WB. K,L) p16 protein (K) and p21 protein (L) in the lung, liver, and spleen excised from 6 month old WT and Mysm1^−/−^ mice (*n* = 3 per group) were measured by IHC. Scale bars, 50 µm. M,N) 2‐month‐old WT and Mysm1^−/−^ mice (*n* = 3 per group) were exposed to IR. p16, MYSM1, and GAPDH protein levels in the lung and liver were determined by WB (M). p16 and p21 protein levels in the lung, liver, and spleen were analyzed by IHC. Scale bars = 50 µm (N). Data are means ± SD. Statistical analyses were performed using an Unpaired *t*‐test in Prism software. *n* = 3 for each sample group. n.s. = no significance.

As senescence is characterized by the extensive secretion of pro‐inflammatory cytokines,^[^ ^40^, ^41^ ^]^ we explored the role of MYSM1 in regulating senescence. ETO‐induced p16 and p21 mRNAs were repressed in MEFs stably expressing Mysm1 (Figure S4A, Supporting Information). ETO‐ or H_2_O_2_‐induced p16 and p21 proteins and mRNAs were significantly higher in Mysm1^−/−^ MEFs (**Figure** **4**A,B and Figure S4B,C, Supporting Information). Notably, ETO‐induced p16 production was suppressed in Mysm1^−/−^ MEFs transfected with Mysm1 (Figure 4C). These data indicate that Mysm1 over‐expression attenuates the production of p16 and p21, whilst Mysm1 knock‐out has the opposite effect.

Our data showed that p16 and p21 levels were significantly higher in Mysm1^−/−^ MEFs relative to WT cells after 10 passages (Figure 4D and Figure S4D, Supporting Information). Interestingly, p16 and p21 mRNAs were higher in the kidney, lung, liver, and spleen of Mysm1^−/−^ mice relative to the WT mice tissues (Figure 4E–H), and p16 protein levels were elevated in the liver and lung of 6 month old Mysm1^−/−^ mice compared to 2 month old Mysm1^−/−^ and WT tissues (Figure 4I,J). Higher levels of p16 and p21 proteins were produced in the lung, liver, and spleen of 6‐month‐old Mysm1^−/−^ mice relative to 2‐month‐old Mysm1^−/−^ and WT mice tissues (Figure 4K,L). Notably, p16 protein levels were induced to very higher levels in the lung and liver of Mysm1^−/−^ mice upon exposure to IR (Figure 4M). Moreover, p16 and p21 protein levels (Figure 4N) and mRNA levels (Figure S4E–G, Supporting Information) were significantly higher in the lung, liver, and spleen of Mysm1^−/−^ mice relative to WT tissues. Collectively, these findings demonstrated that MYSM1 mediated repression of senescence in mice is age‐dependent.

### 2.5 MYSM1 Deficiency Reduces Mice Lifespan

**Figure 5:**
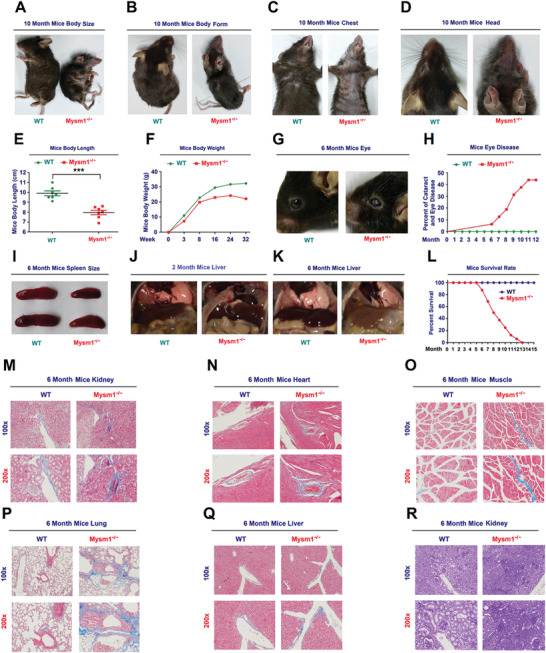
MYSM1 deficiency reduces the lifespan of mice. A–D) Images of the body size (A), body form (B), chest (C), and head (D) of 10 month old WT and Mysm1^−/−^ mice. E) Analysis of body lengths (cm) of 2 month old WT and Mysm1^−/−^ mice (*n* = 7 per group). F) Analysis of body weights of WT and Mysm1^−/−^ mice (*n* = 3 for each time‐point). G) Images of the eyes of 6 month old WT and Mysm1^−/−^ mice. H) Frequencies of cataracts and eye diseases in WT mice (*n* = 16) and Mysm1^−/−^ mice (*n* = 17). I) Images of spleens of 6 month old WT and Mysm1^−/−^ mice (*n* = 5 per group). J) Images of livers of 2 month old WT and Mysm1^−/−^ mice (*n* = 5 per group). K) Images of liver of 6 month old WT and Mysm1^−/−^ mice (*n* = 5 per group). L) Survival rates of WT mice (*n* = 16) and Mysm1^−/−^ mice (*n* = 17). M–Q) Masson's trichrome staining images of kidney (M), heart (N), muscle (O), lung (P), and liver (Q) of 6‐month‐old WT and Mysm1^−/−^ mice (*n* = 3 per group). Scale bars, 100 × = 100 µm, 200 × = 50 µm. R) Periodic acid Schiff (PAS) histochemical staining analyses of 6 month old WT and Mysm1^−/−^ mice kidney (*n* = 3 per group). Scale bars, 100 × = 100 µm, 200 × = 50 µm. Data are means ± SD. Statistical analyses were done by using Prism software by Unpaired t‐tests. ***p < 0.001.

MYSM1 is a key regulator of tissue differentiation and development.^[^ ^15^, ^26^, ^28^ ^]^ We hypothesized that MYSM1 is also involved in the aging process and aging‐related pathologies, as loss of DNA repair associated genes display lymphocyte reduction and tissue decline.^[^ ^34^, ^36^, ^42^, ^43^, ^44^ ^]^ Remarkably, Mysm1^−/−^ mice clearly exhibited aging‐like phenotypes (**Figure** **5**A–D and Figure S5A and Videos S1 and S2, Supporting Information). Unlike WT mice, KO animals had shorter body lengths (Figure 5E) and lower weights (Figure 5F). Mysm1^−/−^ mice developed dull and partially white eyes (Figure 5G), with cataracts and eye diseases developing within 1 to 6 months that rapidly progressing from 6 to 11 months (Figure 5H). Spleen sizes (Figure 5I) and spleen index (mice spleen weight to mice body length) (Figure S5B, Supporting Information) were reduced in Mysm1^−/−^ mice. The livers of 2 month old WT and Mysm1^−/‐^ mice as well as 6 month old WT mice appeared normal and were dark red in color, whereas the livers of 6‐month‐old KO mice were abnormal and were pink in color (Figure 5J,K). Importantly, Mysm1^−/‐^ mice died occurred as early as 5 months, with all Mysm1^−/‐^ animals being dead after 13 months, whereas all WT mice remained alive during the experiment (Figure 5L). Male Mysm1^−/−^ mice died within 5–9 months, whilst female mice died within 5–13 months (Figure S5C,D, Supporting Information). These results confirmed that Mysm1 deficiency facilitates aging, development defects, and early death, reducing the lifespan of mice.

Cells undergoing senescence display functional changes leading to the aging process, which is linked to many pathological changes in the kidneys, such as fibrosis, glomerular sclerosis, and nuclear damage.^[^ ^41^, ^42^ ^]^ In 6‐month‐old Mysm1^−/−^ mice, glomerular sclerosis, tissue destruction, inflammatory infiltration, pyknosis, and renal bleeding were observed in the kidney (Figure S6A, Supporting Information). Fibrosis and cell hypertrophy were observed in the heart (Figure S6B, Supporting Information), and increased atrophy, aging‐associated degradation, muscle fiber disassembly, and tissue infiltration occurred in the muscle (Figure S6C, Supporting Information). Moreover, unlike WT mice, Mysm1^−/−^ mice displayed alveolar epithelial cell proliferation, alveolar septum thickening, and alveolar wall widening in the lung (Figure S6D, Supporting Information). Cell pyknosis, vacuolation, and bleeding were observed in the liver (Figure S6E, Supporting Information), and decreased levels of small lymphocyte, looser cell arrangements, and scattered nodule structure were observed in the spleen (Figure S6F, Supporting Information).

Aged tissue is characterized by increased fibrosis, which leads to aging‐associated diseases.^[^ ^41^ ^]^ Using Masson's trichrome staining to identify fibrotic tissue, we showed clear evidence of fibrosis in the kidney, heart, muscle, lung, and liver of 6 month old KO mice (Figure 5M–Q and Figure S6G, Supporting Information). Periodic Acid Schiff (PAS) staining indicated that glomerular scleroses had developed in the kidney of 6 month old Mysm1^−/−^ mice (Figure 5R). Upon exposure to IR, 6‐month‐old Mysm1^−/−^ mice exhibited abnormal phenotypes, such as alveolar epithelial cell proliferation, alveolar septum thickening, and diffuse lymphocyte infiltration in the lung (Figure S6H, Supporting Information); hepatocyte edema and vacuoles in the liver (Figure S6I, Supporting Information); and decreased lymphocytes, irregular red pulp border, abnormal red‐to‐white pulp ratio, and edema in the spleen (Figure S6J, Supporting Information). Together, the findings demonstrated that Mysm1 deficiency promotes aging‐related pathologies, facilitates developmental defects and early death, thereby shortening the lifespan of mice.

### 2.6 MYSM1 Overexpression Enhances the Lifespan of Mice

The biological roles of MYSM1 in suppressing the aging process and aging‐associated disease were further assessed in mice. Sequencing analysis revealed that human MYSM1 is 98% homologous to monkey MYSM1, and 78% homologous to murine MYSM1 (Figure S7A, Supporting Information). The MYSM1 SANT and SWIRM domains are 100% homologous between monkey and human isoforms. A single amino acid difference was observed between the human and monkey MYSM1 MPN domains, suggesting a high degree of MYSM1 conservation across human, monkey, and murine. The roles of MYSM1 in aging and aging‐associated diseases were determined in mice injected with AAV9‐Ctrl (Figure S7B, Supporting Information) and AAV9‐Mysm1 (Figure S7C, Supporting Information) by two approaches. First, 14 month old WT mice received an intraperitoneal injection of AAV9‐Ctrl (*n* = 4) or AAV9‐Mysm1 (*n* = 5) for 2 months (Figure S7D, Supporting Information). Second, 16 month old WT mice received an intraperitoneal injection of AAV9‐Ctrl (*n* = 5) or AAV9‐Mysm1 (*n* = 5) for 6 months (Figure S7E, Supporting Information).

**Figure 6:**
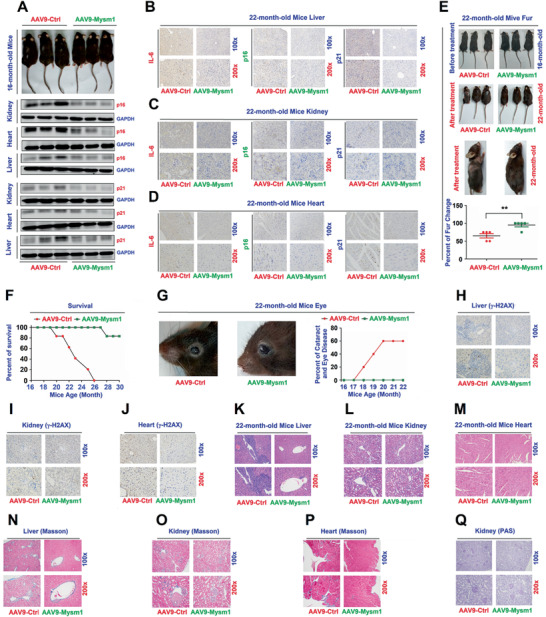
MYSM1 overexpression enhances mice lifespan. A–D) 14‐month‐old WT mice were treated with AAV9‐Ctrl (*n* = 4) or AAV9‐Mysm1 (*n* = 5) for 2 months. Images of 16 month old mice after the treatments (A, top). p16 and p21 proteins in the kidney and heart were measured by WB (a, middle and bottom). IL‐6 (left), p16 (middle), and p21 (right) in the liver (B), kidney (C), and heart (D) of the treated mice were measured by IHC. Scale bars, 100 × = 100 µm, 200 × = 50 µm. E–Q) 16 month old WT mice were treated with AAV9‐Ctrl or AAV9‐Mysml for 6 months (*n* = 5 per group). Images of mice before and after the treatments (E, top), images of mice furs after the treatments (E, middle), and analysis of fur changes in treated mice (E, bottom). Survival rates of 16‐month old WT mice treated with AAV9‐Ctrl or AAV9‐Mysml for 14 months (*n* = 5 per group) (F). Images of the eyes of the treated mice and analysis of frequencies of cataracts and eye diseases (right) (G). *γ*‐H2AX produced in the liver, kidney, and heart of the treated mice were detected by IHC (H–J). H&E stained images of the liver, kidney, and heart of the treated mice (K–M). Masson trichrome staining analyses of the liver, kidney, and heart of the treated mice (N–P). PAS analyses of the treated mice kidney (Q). Scale bars, 10 × = 100 µm, 20 × = 50 µm. Data are presented as the means ± SD. Statistical analyses were performed using Unpaired *t*‐tests in Prism software.

In mice administered with AAV9‐Mysm1, Mysm1 mRNAs were highly expressed in the heart, liver, and kidneys, and were slightly elevated in the lung (Figure S8A,B, Supporting Information). Results from the first approach showed that p16 and p21 proteins were significantly reduced in the kidney and heart of mice treated with AAV9‐Mysm1 (**Figure** **6**A). Similarly, the levels of IL‐6, p16, and p21 proteins decreased in the liver, kidney, and heart of AAV9‐Mysm1‐treated mice (Figure 6B–D). Statistical analysis confirmed that IL‐6, p16, and p21 proteins were significantly reduced in the tissues of AAV9‐Mysm1‐treated mice (Figure S8C–K, Supporting Information).

Hair loss occurred in AAV9‐Ctrl‐treated mice, which was not observed in AAV9‐Mysm1‐treated mice (Figure S9A, Supporting Information). AAV9‐Mysm1 treatment attenuated vacuolar changes, inflammatory infiltration, and glomerular sclerosis in the kidney (Figure S9B, Supporting Information). Decreased inflammatory infiltration was observed in the heart (Figure S9C, Supporting Information), along with reduced cell pyknosis and vacuolation in the liver (Figure S9D, Supporting Information). Statistical analyses conformed that the pathological scores were significantly reduced in the tissues of AAV9‐Mysm1‐treated mice (Figure S9E–G, Supporting Information). Masson's trichrome staining showed that the numbers and size of fibrotic lesions decreased in the tissues of AAV9‐Mysm1‐treated mice (Figure S9H–M, Supporting Information). PAS staining showed that the level of glomerular scleroses was reduced in the kidney of AAV9‐Mysm1‐treated mice (Figure S9N,O, Supporting Information). Together, these results demonstrated that injection of AAV9‐Mysm1 for 2 months resulted in a reduction of the aging process and aging‐associated pathologies in mice.

To confirm the critical role of MYSM1 in the repression of senescence and aging, we carried a second approach, in which 16 month old mice received an intraperitoneal injection of AAV9‐Ctrl or AAV9‐Mysm1 for 6 months. The behaviors of the animals in the two groups before the treatments were observed to be similar and normal (Video S3, Supporting Information). Six months later, the mice in the AAV9‐Ctrl group displayed aged behaviors (Video S4, Supporting Information), whilst the animals in the AAV9‐Mysm group displayed normal behaviors (Video S5, Supporting Information). The degree of hair loss was significantly higher in the AAV9‐Ctrl mice relative to AAV9‐Mysm1 mice (Figure 6E). Remarkably, all AAV9‐Ctrl‐treated mice died between 20 and 26 months, however, only 1 AAV9‐Mysm1‐treated animal died at the age of 28 months. The rest of the AAV9‐Mysm1‐treated mice still survived at ages of 30 months during the experiment (Figure 6F). It was noticed that 3 AAV9‐Ctrl‐treated animals developed dull and partially white eyes, cataracts, and eye diseases at 18, 19, and 20 months of age, while all of the AAV9‐Mysm1‐treated mice showed no signs of eye diseases (Figure 6G).

IHC assays (Figure 6H–J) and statistical analyses (Figure S10A–C, Supporting Information) revealed that *γ*‐H2AX levels were significantly lower in the liver, kidney, and heart of AAV9‐Mysm1‐treated mice relative to the tissues of AAV9‐Ctrl‐treated mice. The levels of vacuolar changes in the vacuole, inflammatory infiltration infiltrations and glomerular scleroses in the kidney, cardiac hypertrophy in the heart, and inflammatory infiltrations and vacuolations in the liver were clearly reduced by AAV9‐Mysm1 (Figure 6K–M). Statistical analyses confirmed that AAV9‐Mysm1 significantly decreased the pathologic scores in the mice tissues (Figure S10D,E, Supporting Information). Masson's trichrome staining showed that number and size of fibrotic lesions were reduced in the liver, kidney, and heart of AAV9‐Mysm1‐treated mice (Figure 6N–P and Figure S10G–I, Supporting Information). Moreover, PAS staining showed that the level of glomerular scleroses was significantly reduced in the kidneys of AAV9‐Mysm1‐treated mice (Figure 6Q and Figure S10J, Supporting Information). These results demonstrated that AAV9‐Mysm1 injection for 6 months significantly repressed the aging process and aging‐associated pathologies in mice.

As DNA damage can induce apoptosis,^[^ ^31^ ^]^ we checked the levels of apoptosis in mice tissues using TUNEL straining. Higher numbers of TUNEL‐positive cells were observed in the liver of AAV9‐Ctrl‐treated mice compared to AAV9‐Mysm1‐treated mice (Figure S11A, Supporting Information), confirming that MYSM1 suppresses DNA damage‐induced senescence and apoptosis. Immune cell changes in mice tissues were further assessed through IHC. Importantly, higher levels F4/80 (a macrophage maker) positive cells were detected in the liver and kidney of AAV9‐Ctrl‐treated mice liver and kidney as compared to AAV9‐Mysm1‐treated mice tissues (Figure S11B,C, Supporting Information). However, there were no differences were observed in the numbers of CD3 (a T cell marker) positive cells between AAV9‐Ctrl‐ and AAV9‐Mysm1‐treated mice (Figure S11D,E, Supporting Information). In addition, the levels SA‐*β*‐Gal positive cells were higher in the kidney of AAV9‐Ctrl‐ compared to AAV9‐Msym1‐treated mice kidney (Figure S11F, Supporting Information). These results suggested that AAV9‐Mysm1 treatment suppresses inflammatory cell infiltration, and has no effect on lymphocyte differentiation in mice organs.

**Figure 7:**
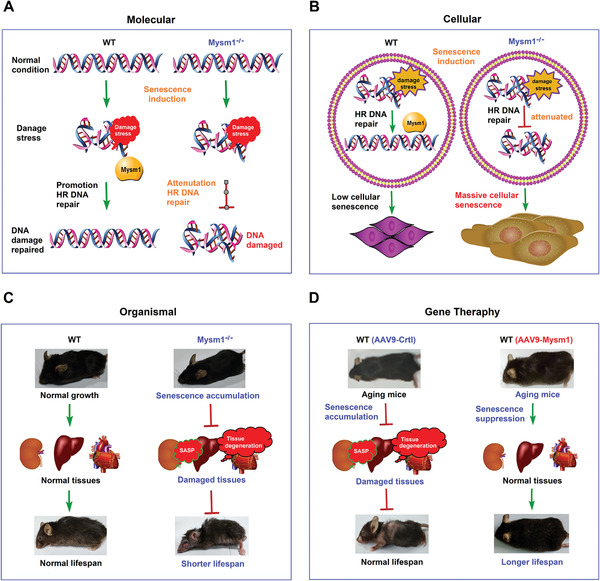
MYSM1 is a key suppressor of aging and aging‐related pathology. A) The role of MYSM1 in promoting DNA repair. B) The function of MYSM1 in repressing cellular senescence and the aging process. C) MYSM1 deficiency facilitates aging, promotes aging‐related pathology, and reduces the lifespan of mice. D) MYSM1 over‐expression attenuates the aging process, reduces aging‐related pathology, and extends the lifespan of mice.

Our data revealed that MYSM1 is a key suppressor of aging and aging‐related pathologies. MYSM1 functionally represses cellular senescence and the aging process by promoting DNA repair. MYSM1 deficiency facilitates the aging process and reduces lifespan, whereas MYSM1 over‐expression attenuates aging and increases the lifespan of mice (**Figure** **7**).

## 3 Discussion

Cellular senescence is a state in which cells undergo irreversible cell cycle arrest upon many stresses and stimuli.^[^ ^43^, ^45^ ^]^ Senescent cells accumulate during the aging process that may promote aging and aging‐associated diseases.^[^ ^46^ ^]^ Aging is characterized by a progressive loss of lifespan, leading to impaired tissue function and increased vulnerability to death. This deterioration is a major risk factor for many human diseases, including cancer, diabetes, cardiovascular disorders and neurodegenerative diseases.^[^ ^47^ ^]^ Although many intracellular pathways have been reported to regulate the aging process, the upstream events controlling aging require further characterization.

This study reported a key role for MYSM1 in the restriction of cellular senescence, aging process, and aging‐associated diseases. Initially, we showed that MYSM1 expression is promoted in the early stages of senescence induction, whilst its production is decreased in the later stages of senescence in both senescent cells and aged mice. These results suggested that MYSM1 is highly associated with cellular senescence and aging. Detailed functional analyses confirmed that MYSM1 represses cellular senescence and the aging process in human and mice primary cells and in mice organs.

Whilst evaluating the molecular mechanisms underlying the MYSM1‐mediated aging process, we revealed that MYSM1 represses cellular senescence and aging process by facilitation of HR DNA repair. DNA damage is as a key factor during senescence and aging,^[^ ^31^, ^32^, ^33^, ^34^, ^35^, ^36^, ^37^ ^]^ here we showed that in response to intracellular senescence, MYSM1 overexpression down‐regulates the production of senescence‐associated cytokines, whereas MYSM1 knockout up‐regulates cytokine production. In addition, Mysm1 deficiency led to the induction of SASP in mouse tissues. These data suggested that MYSM1 plays an inhibitory role in SASP‐associated signaling.

Components of the Rb and p53 pathways are biomarkers of senescence.^[^ ^38^ ^]^ Induction of Rb^[^ ^48^, ^49^ ^]^ and p53 pathways,^[^ ^50^, ^51^ ^]^ in senescent cells facilitates permanent cell cycle arrest, aged cell accumulation, tissue degeneration, and the development of diseases. MYSM1 restricts the Rb and p53 pathways in senescent cells and mouse tissues, improves tissue function, and reduces the accumulation of aged cells. Our data revealed a distinct mechanism through which MYSM1 eliminates DNA damage by promoting DNA repair to suppress senescence and the aging processes.

The biological roles of MYSM1 in suppressing the aging process were further determined and confirmed by two critical approaches: knock‐out of MYSM1 and over‐expression of MYSM1 in mice. MYSM1 knock‐out mice showed aging‐like phenotypes manifested as shorter body lengths, lower body weights, cataracts and eye diseases, smaller spleen, and abnormal livers. Remarkably, KO mice died as early as 5 months of age, whereas WT mice survived the course of the experiment. Aged tissue is characterized by increased fibrosis, which leads to aging‐associated diseases.^[^ ^47^ ^]^ We provided strong evidence of fibrotic lesions in the kidney, heart, muscle, lung, and liver of 6‐month‐old KO mice. These findings demonstrated that Mysm1 deficiency resulted in the promotion of aging, aging‐related pathology, development defects, and early death in mice.

The roles of MYSM1 in aging and aging‐associated diseases were determined by injection of WT mice with AAV9‐Mysm1. In 14 month old WT mice who received an intraperitoneal injection of AAV9‐Mysm1 for 2 months, the levels of p16 and p21 proteins were significantly reduced in the liver, kidney, and heart. Hair loss was not observed; and the pathology scores were significantly reduced in the kidney, heart, and liver. 16 month old WT mice who received an intraperitoneal injection of AAV9‐Mysm1 for 6 months displayed normal behaviors, whilst the control mice displayed aged behaviors. Remarkably, all untreated mice died at 20 to 26 months, whilst only 1 AAV9‐Mysm1‐treated animal died at 28 months and 80% AAV9‐Mysm1‐treated animals survived at 30 months. Notably, AAV9‐Mysm1 treatment significantly reduced levels of DNA damages and the pathologic scores in mice. Together, these results demonstrated that injection of AAV9‐Mysm1 led to the repression of aging process and aging‐associated pathologies, and thus enhancing lifespan in mice.

In summary, we identified MYSM as a key suppressor of senescence, aging, and aging‐associated diseases. MYSM1 represses the processes of senescence and the progress of aging, prevents the damage to vital organs, and reduces the development of aging‐associated diseases, which lead to the prolongation of lifespan. We believe that MYSM1 plays important roles in many biological processes beyond those investigated herein, which makes MYSM1 as a potential agent for the prevention of aging and the development of age‐associated diseases.

## 4 Experimental Section

##### Animal Experiments

C57BL/6 WT mice were purchased from the Hubei Research Center of Laboratory Animals (Wuhan, China). MYSM1^−/‐^ mice [Mysm1_A04; Mysm1tm1a(ΔMP)Wtsi MGI#:2 444 584] on the C57BL/6J background were a kind gift of Dr. Xiaoxia Jiang of the Institute of Military Cognition and Brain Sciences, the Academy of Military Medical Sciences, Beijing, China. All animal studies were performed following the principles described by the Animal Welfare Act and the National Institutes of Health Guidelines for the care and use of laboratory animals in biomedical research. All procedures involving mice and experimental protocols were approved by the Institutional Animal Care and Use Committee (IACUC) of the College of Life Sciences, Wuhan University, Wuhan, China.

##### Ionizing Radiation

For ionizing radiation exposures, mice were subjected to a sub‐lethal dose of 4 Gy, and tissues harvested at 7 days after the exposure for immunohistochemistry and quantitative real‐time PCR (qRT‐PCR) analyses.

##### Preparation of Mouse Specimens

Total RNA was extracted from 50 mg of different tissues. Tissues were first minced and then homogenized using the TRIzol reagent (Invitrogen). After phase separation, the aqueous layer containing RNA was processed with a Purelink RNA Mini Kit (Zhuangmeng, Beijing) to yield total RNA. For real‐time quantitative PCR (RT‐qPCR) experiments, 1 µg of this RNA was reverse transcribed into cDNA. In each experiment, samples were all prepared in parallel without blinding.

For immunohistochemistry (IHC) assessment of tissues, samples were fixed using 4% paraformaldehyde, paraffin‐embedded, sliced into these sections, and attached to microscope slides. IHC staining was performed based on standard procedures.

For hematoxylin and eosin (H&E) staining, multiple sections of each organ were analyzed, with investigators blinded to the genotypes of each sample. Tissue pathological scores were assigned by evaluating 3 random fields of view per mouse, which were scored as follows: 0, absence of damage; 1, <15% tissue area damaged; 2, 15–50% tissue area damaged; 3, >50% tissue area damaged. The scores from three fields were added together to give a final score for each animal. For statistical analysis of IHC images, 3–5 fields of view were selected in a double‐blinded manner, and image analysis performed using Image J.

Masson Trichrome staining was performed according to the manufacturer's instructions and image analysis performed using Image J. The presence of sclerosis was assessed based on the staining intensity and size of Periodic Acid‐Schiff (PAS)‐positive lesions within the glomeruli.

##### Fur Change Analysis

Aging mice displayed reduced fur density. Fur changes were scored on a scale of 0–4 point, where 0 was very lower fur and 4 was normal fur. Each mouse was scored before treatment and after treatment. The final percentage was determined as the ratio score (before treatment)/(after treatment). The percentage of changes was subsequently plotted.

##### Apoptosis and Immune Cell Analyses

Apoptosis and change in immune cells were determined by Immunohistochemistry (IHC). TUNEL staining in the liver of mice was used to determine the level of apoptosis. For statistical analysis of IHC images, 3–5 fields of view were selected in a double‐blinded manner, and the images were analyzed using Image J. F4/80 and CD3 staining were used to analyze immune cell infiltrations. For statistical analysis of IHC images, 3–5 fields of view were selected in a double‐blinded manner, and the images were analyzed using Image J.

##### AAV9‐Mysm1 Construction and Treatment

The AAV9 delivery system was used to express Mysm1 in mice. AAV9‐Ctrl and AAV9‐Mysm1 constructs were generated by Heyuan Biology (Shanghai, China). The vector AOV021 pAAV9‐CMV‐MCS‐3FLAG was used as the control (AAV9‐Ctrl). To generate AAV9‐Mysm1 construct, the full‐length DNA fragment of the mouse Mysm1 gene (GenBank ID: NM_177 239) was amplified by PCR using cDNA isolated from murine embryonic fibroblasts (MEFs) of C57BL/6 WT mice as the template. The resulting DNA fragment of the mouse Mysm1 gene was then inserted into *Nhe*I and *Bam*HI restriction sites of the vector AOV021 pAAV9‐CMV‐MCS‐3FLAG to generate AAV9‐Mysm1. The sequences of the insert (mouse Mysm1 gene) in the resulting construct AAV9‐Mysm1 were confirmed by sequencing analysis using the sense primer (CMV‐F CGCAAATGGGCGGTAGGCGTG) and anti‐sense primer (WPRE‐R CATAGCGTAAAAGGAGCAACA).

In the experiments, C57BL/6 WT mice purchased from the Hubei Research Center of Laboratory Animals (Wuhan, China) were bred in the animal center of Wuhan University. 14 month old mice received an intraperitoneal injection of AAV9‐Ctrl or AAV9‐Mysm1 (2.5 × 10^11^ MOI) and 2 months later the treated mice were used for appropriate experimental analyses. In a second approach, 16 month old mice received an intraperitoneal injection of AAV9‐Ctrl or AAV9‐Mysm1 (2.5 × 10^11^ MOI), and 6 months later the treated mice were used for appropriate experimental analyses.

##### Ethics Statement

This study was conducted according to the principles of the Declaration of Helsinki and approved by the Institutional Review Board of the College of Life Sciences, Wuhan University, in accordance with guidelines for the protection of human subjects. The Institutional Review Board of the College of Life Sciences, Wuhan University, approved the collection of blood samples for this study. All procedures were conducted in accordance with the guidelines for the protection of human subjects. Written informed consent was obtained from each participant.

All animal studies were performed in accordance with the principles of the Animal Welfare Act and the National Institutes of Health Guidelines for the care and use of laboratory animals in biomedical research. All procedures involving mice and experimental protocols were approved by the Institutional Animal Care and Use Committee (IACUC) of the College of Life Sciences, Wuhan University (Permit numbers: 2019‐009).

##### Cell Lines and Cultures

Human diploid fibroblasts (WI‐38) were purchased from the American Type Culture Collection (ATCC, #CRL‐7728; Manassas, VA, USA). Murine embryonic fibroblasts (MEFs) were isolated from the fetuses of pregnant C57BL/6 WT mice or pregnant C57BL/6 Myms^−/−^ mice. MEFs were cultured in Dulbecco's modified Eagle's medium (DMEM) (Gibco, Grand Island, NY, USA) containing 10% Fetal Bovine Serum (FBS), 100 U mL^−1^ penicillin and 100 µg mL^−1^ streptomycin sulfate.

In experiments involving drug treatments, cells were grown until 60–70% confluent, and then treated for 48 h with either etoposide (0–40 × 10^−6^ m) or doxorubicin (0–1 × 10^−6^ m) for 48 h. Culture media were exchanged, and cells were collected on day 7. In experiments assessing hydrogen peroxide (H_2_O_2_)‐induced senescence, cells were grown to 60–70% confluency and then treated with H_2_O_2_ (0–200 × 10^−6^ m) for 2 h. Culture media were then replaced, and cells were collected on day 7.

In experiments assessing replicative senescence, MEFs were treated as follows: torsos from 13.5 day old embryos were culture in media containing 10% Fetal Bovine Serum (FBS), 100 U mL^−1^ penicillin and 100 µg mL^−1^ streptomycin sulfate. After 2 days, cells grown from tissue fragments were transferred into 75 cm^2^ flasks and cultured to 90% confluency. From the enriched fibroblast population, 5 × 10^5^ cells were sub‐cultured in 10 cm dishes and considered as passage 1 and 1P.

For population doubling experiments, when cultures were confluent, cells were passaged by trypsinization and the cell number counted. The added number of population doublings during each passage was calculated by the equation *A* = log2*H* − log2*S* (*A*, added population doublings; *H*, cell number at the end of each passage; *S*, cell number at the beginning of each passage).

##### Antibodies and Reagents

Antibodies against Flag (F3165) (1:2000) and monoclonal mouse anti‐GAPDH (G9295) (1:5000) were purchased from Sigma (St Louis, MO, USA). Anti‐P21 (sc‐6246 Santa Cruz), anti‐P16^ink4a^ (sc‐166760 Santa Cruz), anti‐P16^ink4a^ (abs130164 Absin) and anti‐MYSM1 (Abcam, ab‐193081) (1:100) were purchased from the indicated manufacturers. Anti‐*γ*‐h2ax was purchased from Abcam.

Lipofectamine 2000, normal rabbit immunoglobulin G (IgG), and normal mouse IgG were purchased from Invitrogen (Carlsbad, CA, USA). Etoposide, and doxorubicin were purchased from Sigma (St Louis, MO, USA).

##### Plasmids and Transfection

The full‐length DNA fragment of human Myms1 gene was amplified by PCR using cDNA isolated from human acute monocytic leukemia cells (THP‐1) as the template. The full‐length DNA fragment of the mouse Mysm1 gene was amplified by PCR using cDNA isolated from MEFs of C57BL/6 WT mice as the template. The resulting human or mouse Mysm1 genes was respectively inserted into mammalian expression vectors, as indicated. Plasmid transfections were performed using Lipofectamine 2000 (Invitrogen) according to the manufacturer's instructions.

##### Sequencing Analyses

The sequences of Mysm1 genes isolated from human, monkey (macaque) and mouse were download from NCBI. Alignment was generated using Cluste X, and the conserved residues then analyzed by Genedoc. Identical residues are indicated by the yellow background and red words, and the blue background indicated two identical residues that are different from another residues.

##### Western Blotting

Cellular proteins were isolated by lysing cells with lysis buffer (50 × 10^−3^ m Tris‐HCl, pH7.5, 0.5 × 10^−3^ m EDTA, 150 × 10^−3^ m NaCl, 1% NP40, 1% SDS) containing 1:100 Protease and phosphatase inhibitor cocktail (Roche). Lysed samples were then agitated for 1 h at 4 °C, and the supernatants were collected, boiled in protein loading buffer for 5 min, and separated via SDS‐PAGE. Samples were then transferred onto nitrocellulose membranes blocked with 5% milk in Tris buffered saline with Tween‐20 (TBST) at RT for 1 h. Blots were then probed overnight at 4 °C with primary antibodies diluted in 5% bovine serum albumin (BSA) in TBST. Blots were washed three times for 10 min in TBST, and then probed with an appropriate HRP‐conjugated secondary antibody for 1 h. After additional washing, blots were imaged using X‐ray film or a LAS‐4000 Imager.

##### RT‐PCR

Trizol Reagent was used to extract total RNA from tissues or cells harvested with 0.25% trypsin ethylenediaminetetraacetic acid (EDTA) (1x, Gibco Grand Island, NY, USA). cDNA was generated with M‐MVL reverse transcriptase. For RT‐PCR reactions, specific primers and iTaq SYBR Green Fast qPCR Master Mix (DBI Bio‐science, Ludwigshafen, Germany) were used, with samples analyzed in triplicate using glyceraldehyde‐3‐phosphate dehydrogenase (GAPDH) as a normalization control. The PCR cycling conditions were as follows; 42 °C for 5 min, 95 °C for 10 s, and 40 cycles of 95 °C for 5 s, and 60 °C for 30 s. The PCR primers used in this study are listed in Table S1 (Supporting Information).

##### Immunofluorescence and Confocal Microscopy

MEFs were cultured in confocal culture dish for 24 h and fixed with 4% paraformaldehyde for 15 min. After washing three times with PBS, cells were permeabilized with 0.1% Triton X‐100 for 15 min and then washed again with PBS for 3 times. Blocking was performed in 5% BSA for 45 min. Dishes were incubated with 1% BSA or PBS containing primary antibodies for 2 h or overnight at 4 °C, followed by four washes in PBS to remove unbound primary antibodies. Samples were incubated with Secondary antibodies (FITC and Dylight‐649) for 1 h and then wash three time with PBS. DAPI (1 µg mL^−1^) was applied for 10 min before washing thoroughly for 4 times in PBS. Images were acquired on an Olympus FV1000 fluorescence microscope and analyzed using Image J. At each time point, 3–8 pictures were analyzed, which contained more than 50 cells.

##### Enzyme Linked Immunosorbent Assay (ELISA)

ELISA kit (BD Biosciences, San Jose, CA, USA) was used to determine the level of IL‐6 protein in the cell supernatants or sera. The experimental procedures were performed according to the manufacturer's instructions.

##### Senescence‐Associated *β*‐galactosidase Assay

Senescence‐associated *β*‐galactosidase (SA‐*β*‐Gal or SA*β*G) staining was performed according to the manufacturer's instructions (Biovision, no. k320‐250). Samples were incubated overnight with a*β*‐gal detection solution at 37 °C, and quantified using a regular light microscopy.

##### Analysis of Homologous Recombination and Nonhomologous End Joining

Human HEK293T cells and mice MEFs were seeded in 6‐well plates, cells were grown until 60–70% confluency. Cells The cells were then transfected with the I‐SceI linearized homologous recombination (HR) or non‐homologous end joining (NHEJ) reporter, together with pCMV‐DsRed. Three days after transfection, cells were collected for analysis of DNA repair efficiency by flow cytometry (FCM). The efficiency of HR or NHEJ was determined as the ratio of GFP+ and DsRed+ cells, respectively. At least 10 000 cells were counted and experiments were performed in triplicate.

##### Cell Viability and Proliferation Assays

Cells were plated in triplicate in 96‐well plates. Cell viability and proliferation were assessed at time points after plating using the CELL COUNTING kit‐8 (DOJINDO). Staining was performed according to the manufacturer's instructions.

##### Colony Formation and Cell Density Assays

Etoposide‐ and doxorubicin‐induced senescence was assessed in primary MEFs after cells were plated in 6‐well plates in triplicate for 1 week. Cells were fixed in methanol and stained with 0.5% crystal violet in 25% methanol. Plates were dried, and the cell density quantified by de‐staining in 10% acetic acid, and the absorbance measured at 590 nm.

##### Statistical Analysis

Statistical analysis was performed in Graphpad Prism 5. Data are presented as the means ±SD. For cell experiments, *n* = 3 for per group, for mice experiment, each group from number 2–10. Details are shown in the Figure Legends. Samples were compared using the student's t‐test and ANOVA with Prism 5 (GraphPad Software Inc.). *p* ≤ 0.05 was the significance threshold. ns, not significant (*p* > 0.05); *, *p* < 0.05, **, *p* < 0.01 and ***, *p* < 0.001).

## Conflict of Interest

The authors declare no conflict of interest.

## Author Contributions

M.T., Y.H., P.Z., W.L., Y.S., W.L., K.W., and J.W. contributed to the design of experiments. M.T., Y.H., P.Z., W.L., Y.S., W.L. contributed to the conduction of experiments. M.T., Y.H., P.Z., W.L., Y.S., W.L., K.W., and J.W. contributed to the reagents. M.T., Y.H., and J.W. contributed to the writing the paper. M.T. and J.W. contributed to the editing the paper. M.T. and Y.H. contributed equally to this work.

## Supporting information

Supporting Information

Supplemental Video 1

Supplemental Video 2

Supplemental Video 3

Supplemental Video 4

Supplemental Video 5
