# Supplementary material for: MYSM1 Suppresses Cellular Senescence and the Aging Process to Prolong Lifespan
Source: Adv Sci (Weinh). 2020 Sep 30;7(22):2001950. doi: 10.1002/advs.202001950 (PMC7675055; doi:10.1002/advs.202001950)
Supplement: Supplementary file 1 — Supporting Information [file ADVS-7-2001950-s001.pdf]

© 2020 Wiley-VCH GmbH

Supporting Information

for *Adv. Sci.,* DOI: 10.1002/advs.202001950

MYSM1 Suppresses Cellular Senescence and the Aging Process to Prolong Lifespan

*Mingfu Tian, Yuqing Huang, Yunting Song, Wen Li, Peiyi Zhao, Weiyong Liu, Kailang Wu, and Jianguo Wu**

MYSM1 suppresses cellular senescence and the aging process to prolong lifespan

Supplementary Information

Supplementary Figures and Legends

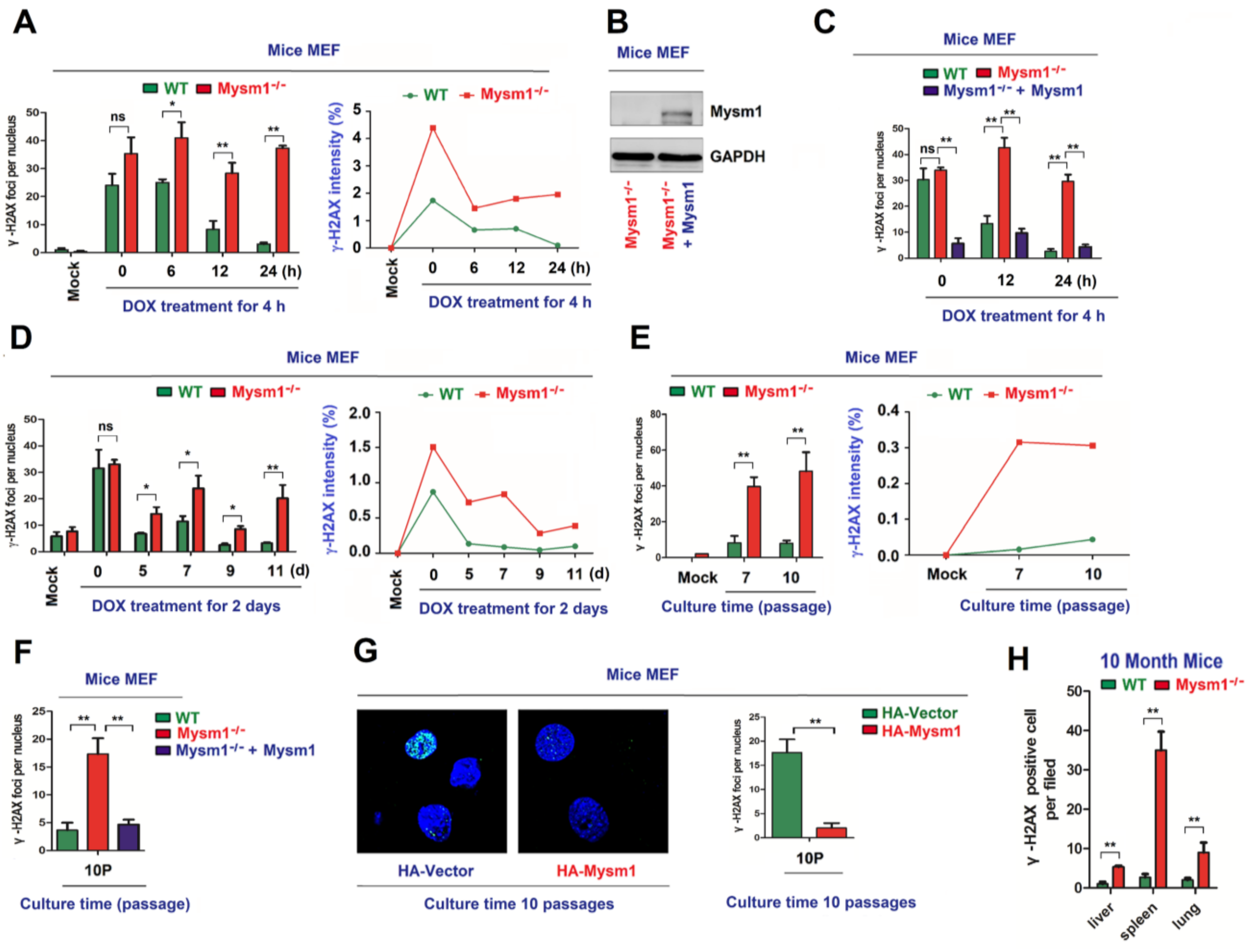

Figure S1. MYSM1 promotes HR DNA repair. (**A**) MEFs of WT mice or Mysm1$^{-/-}$ mice were treated with DOX for different time as indicated. Statistical analysis of γ-H2AX levels in DOX-treated MEFs. (**B** and **C**) Mysm1$^{-/-}$ MEFs were transfected with plasmid expressing Mysm1. MYSM1 protein in Mysm1$^{-/-}$ MEFs and Mysm1$^{-/-}$ MEFs transfected with plasmid expressing Mysm1 were assessed by WB (B). Statistical analysis of γ-H2AX strain levels in DOX-treated WT MEFs, Mysm1$^{-/-}$ MEFs, and Mysm1$^{-/-}$ MEFs transfected with Mysm1 (C). (**D**) Statistical analysis of γ-H2AX strain levels in WT and Mysm1$^{-/-}$ MEFs treated with DOX for 2 days and then grown for different times as indicated. (**E**) Statistical analysis of γ-H2AX strain levels in WT and Mysm1$^{-/-}$ MEFs continuously grow for different passages as indicate. (**F**) Statistical analysis of γ-H2AX strain

levels in WT MEFs, Mysm1$^{-/-}$ MEFs, and Mysm1$^{-/-}$ MEFs transfected with Mysm1 after continuously growth for 10 passages. (**G**) WT mice MEFs transfected with Flag-Vector or Flag-Mysm1 were and then of were grown continuously continuously growth for 10 passages. The γ-H2AX levels in the MEFs were detected and visualized under confocal microscope (left), and statistical analysis of γ-H2AX strain levels in the MEFs were measured by image J (right). (H) Liver, spleen, and lung were excised from 10-month-old WT and Mysm1$^{-/-}$ mice (n=3 per group). γ-H2AX abundances were determined by IHC, and statistical analysis of γ-H2AX strain levels in the MEFs were measured by image J. Data are means ±SD. Statistical analyses were done by using Prism software by Unpaired t-tests. *$p<0.05$, **$p<0.01$, ***$p<0.001$, n.s =no significance; n=3 for each sample group.

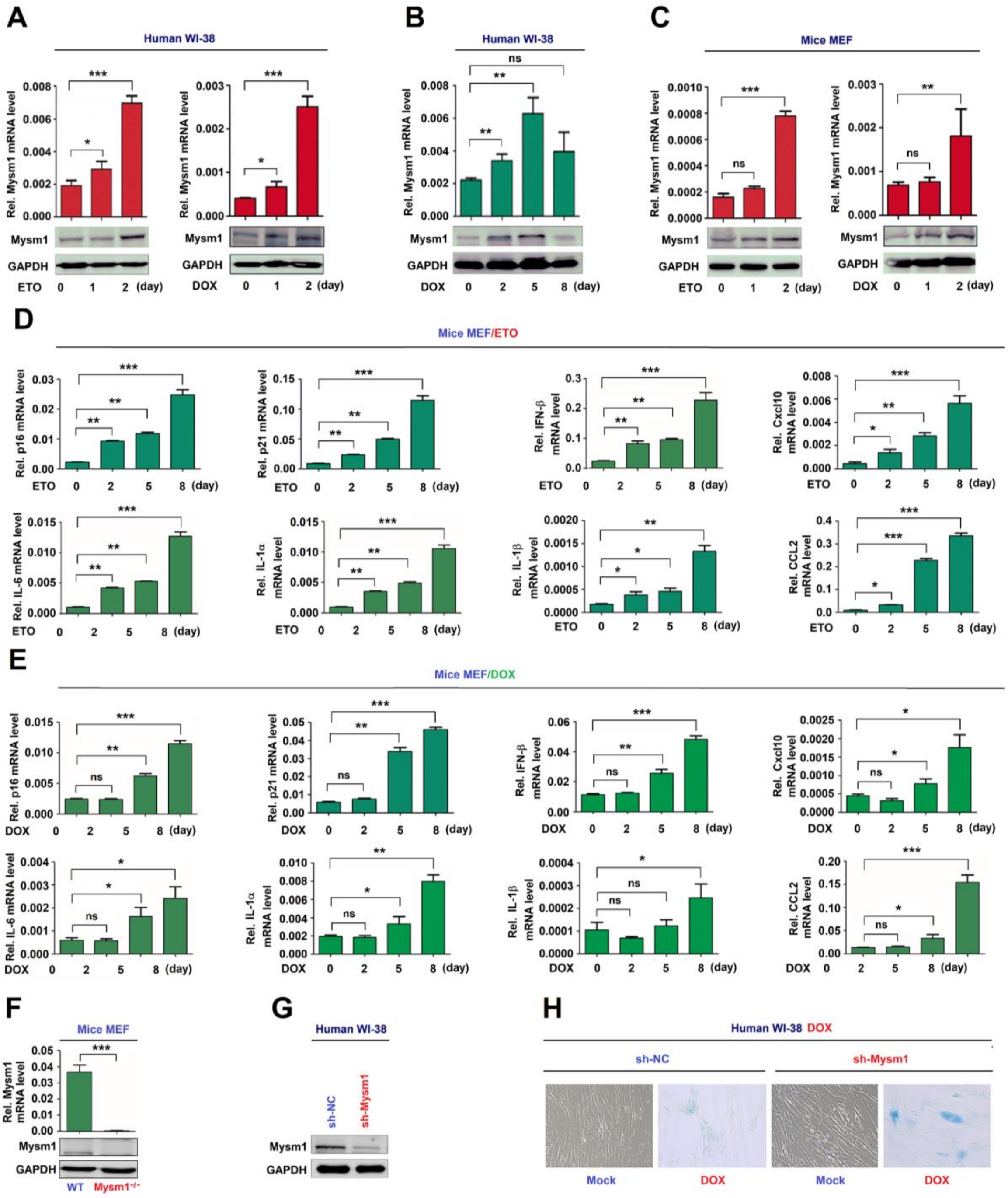

Figure S2. MYSM1 correlates with senescence process. (**A**–**C**) Human WI-38 cells were treated with Etoposide (ETO) and Doxorubicin (DOX) for different times as indicated (A and B). MEFs of C57BL/6 mice were treated with DOX and ETO for 2 days (C). Mysm1 mRNA levels were assessed by RT-PCR (top) and Mysm1 protein levels were detected by Western blot analysis (WB) (bottom). (**D** and **E**) MEFs of C57BL/6 mice were treated with ETO (D) and DOX (E) for different days as

indicated to induced cellular senescence. The mRNA levels of senescence-associated cytokines, including p16, p21, IFN-β, Cxcl10, IL-6, IL-1α, IL-1β, and CCL2, were assessed by RT-PCR. (**F**) Mysm1 mRNA and MYSM1 protein in WT or Mysm1$^{-/-}$ MEFs were analyzed by RT-PCR and WB, respectively. (**G**) MYSM1 protein in human WI-38 cells transfected with sh-NC or sh-Mysm1 were determined by WB. (**H**) Senescence-associated β-galactosidase (SA-β-Gal) activities were determined in human WI-38 cells stably expressing sh-NC or sh-Mysm1 treated with DOX. Data are means ±SD. Statistical analyses were done by using Prism software by Unpaired t-tests. *p<0.05, **p<0.01, ***p<0.001, n.s = no significance; n = 3 for each sample group.

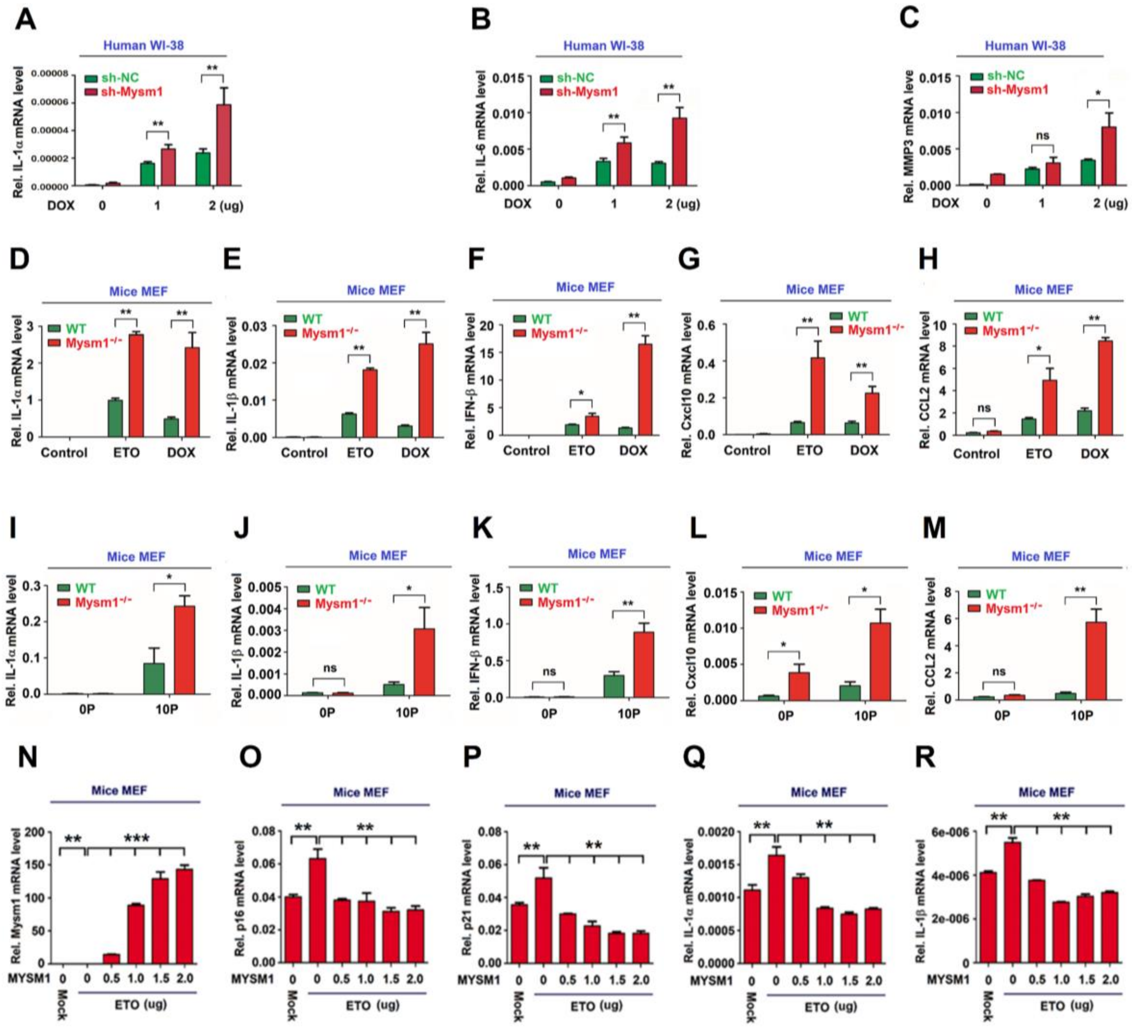

Figure S3. MYSM1 suppresses DDR-associated SASP. (**A**–**C**) Human WI-38 cells stably expressing sh-Mysm1 or sh-NC were generated, and then treated with DOX. The IL-1α mRNA (A), IL-6 mRNA (B), and MMP3 mRNA (C) expressed in the cells were quantified by RT-PCR. (**D**–**M**) WT and Mysm1$^{-/-}$ mice MEFs were treated with ETO or DOX (D–H), or continuously growth for 10 passages (I–M). The mRNA levels of SASP-associated cytokines, including IL-1α (D and I), IL-1β (E and J), IFN-β (F and K), Cxcl10 (G and L), CCL2 (H and M), were quantified by RT-PCR. (**N**–**R**) WT mice MEFs were transfected with Flag-Mysm1 at different concentrations as indicated. The cells were treated with ETO and the mRNA levels of Mysm1 and SASP-associated cytokines, including

p16, p21, IL-1α, and IL-1β, were assessed by RT-PCR. Data are means ±SD. Statistical analyses were done by using Prism software by Unpaired t-tests. *$p<0.05$, **$p<0.01$, ***$p<0.001$, n.s = no significance; n = 3 for each sample group.

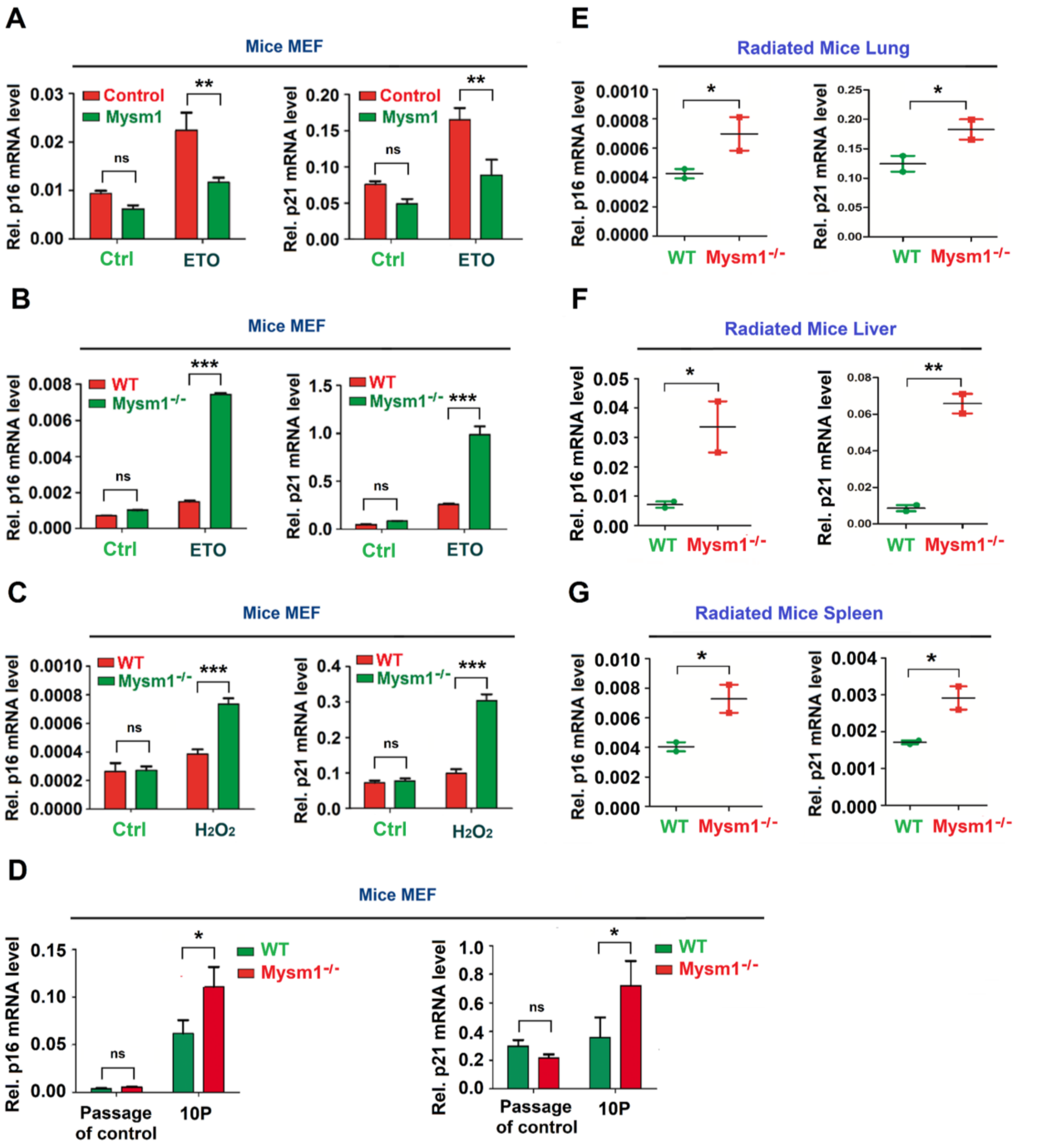

Figure S4. MYSM1 represses senescence in aged mice. (**A**–**D**) WT mice MEFs stably expressing Mysm1 were generated and then treated with ETO (A). WT and Mysm1$^{-/-}$ mice MEFs were treated with ETO (B) or H_2O_2 (C, or continuously growth for 10 passages (D). The mRNA levels of p16 (left) and p21 (right) expressed in treated MEFs were quantified by RT-PCR. (**E**–**G**) WT and Mysm1$^{-/-}$ mice were subjected to IR. The mRNA levels of p16 (left) and p21 (right) expressed in the

lung (E), liver (F), and spleen (G) of the treated mice were quantified by RT-PCR. Data are means ± SD. Statistical analyses were done by using Prism software by Unpaired t-tests. *$p < 0.05$, **$p < 0.01$, ***$p < 0.001$, n.s. = no significance; n = 3 for each sample group (A–D), n = 2 for each sample group (E–G).

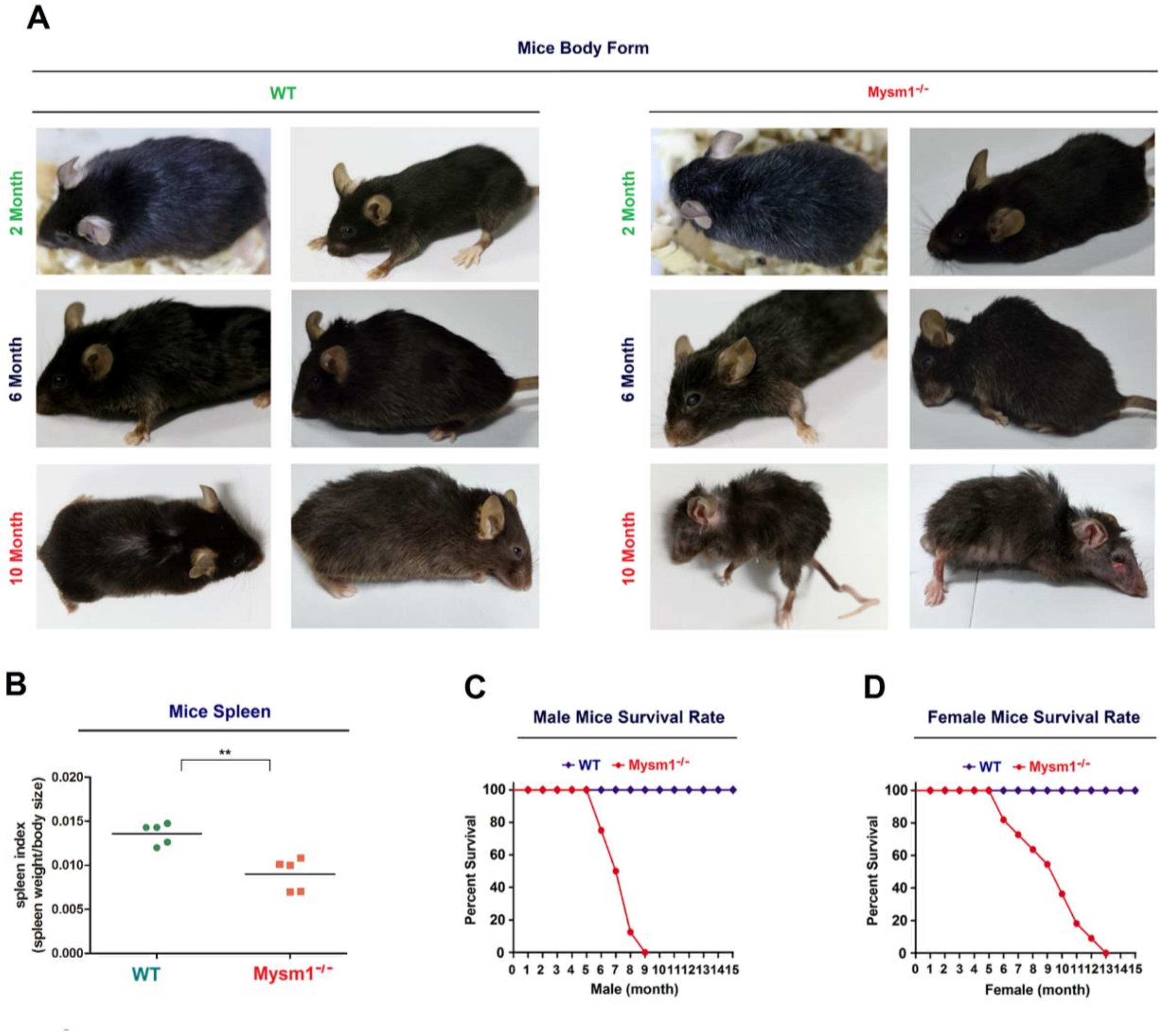

Figure S5. Mysm1 deficiency reduces mice lifespan. (**A** and **B**) WT mice (n=16) and Mysm1$^{-/-}$ mice (n=17) were reared under identical conditions. Representative images of the body forms of mice at 2, 6, and 10 months of age are shown (A). Spleen index (spleen size to body length) of WT mice and Mysm1$^{-/-}$ mice were measured (B). (**C** and **D**) WT mice (n = 16; 8 males, 8 female) and Mysm1$^{-/-}$ mice (n = 19; 8 males, 11 female) were reared under identical conditions for 15 months. The survival rate of male mice (C) and female mice (D) were assessed on a monthly basis. Data are means ± SD. Statistical analyses were done by using Prism software by Unpaired t-tests. *$p < 0.05$, **$p < 0.01$, ***$p < 0.001$, n.s. = nonsignificant; n = 5 for each sample group.

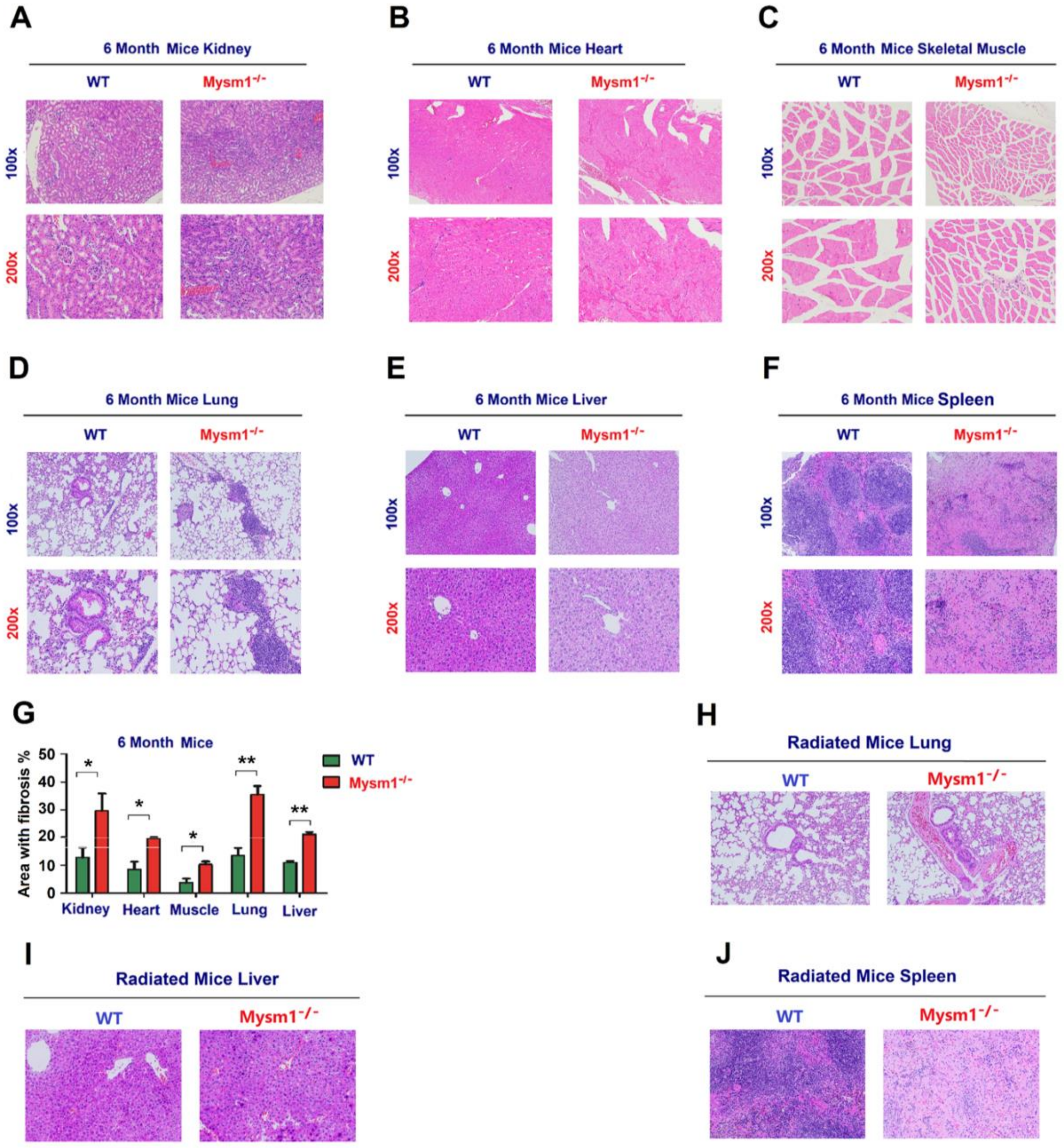

Figure S6. Mysm1 deficiency promotes aging-related disease. (**A**–**F**) H&E-stained images of the kidney (A), heart (B), muscle (C), lung (D), liver (E), and spleen (F) of 6-month old WT and Mysm1$^{-/-}$ mice (n = 3 per group). (**G**) Statistical analysis of fibrotic lesions in the kidney, heart, muscle, lung, and liver of 6-month-old KO mice. (**H**–**J**) H&E staining of the lung (H), liver (I), and spleen (J) of 2-month old WT mice and Mysm1$^{-/-}$ mice exposed to IR (n = 3 per group) were performed. Data are means ± SD. Statistical analyses were done by using Prism software by

Unpaired t-tests. *$p < 0.05$, **$p < 0.01$, ***$p < 0.001$, n.s. = no significance; n = 3 for each sample group.

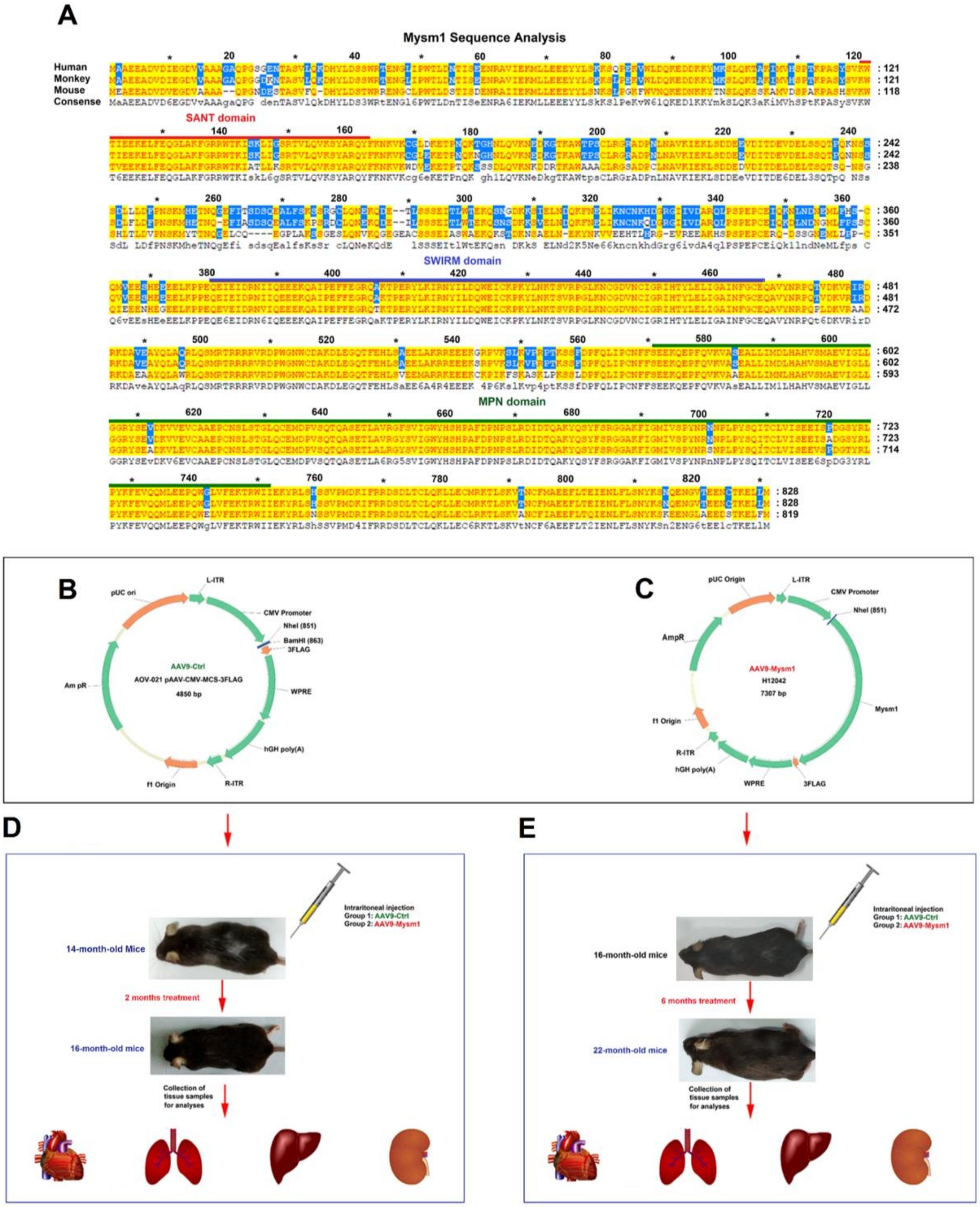

Figure S7. AAV9-Mysm1 treatment. (**A**) MYSM1 sequences from humans, monkeys (macaques), and mice were downloaded from NCBI. Cluste X was used to align these sequences, and Genedoc was used to identify conserved residues. Identical residues are indicated by a black background, while conserved residues have a dark background. (**B** and **C**) The diagrams of constructed AAV9-

Ctrl (B) and AAV9-Mysm1 (C). (**D** and **E**) 14-month old WT C57BL/6 mice were received an intraperitoneal injection of AAV9-Ctrl or AAV9-Mysm1 (2.5 x 10^{11} MOI) for 2 months (D). 16-month-old WT C57BL/6 mice were received an intraperitoneal injection of AAV9-Ctrl or AAV9-Mysm1 (2.5 x 10^{11} MOI) for 6 months (E). The treated mice were used for appropriate experimental analyses.

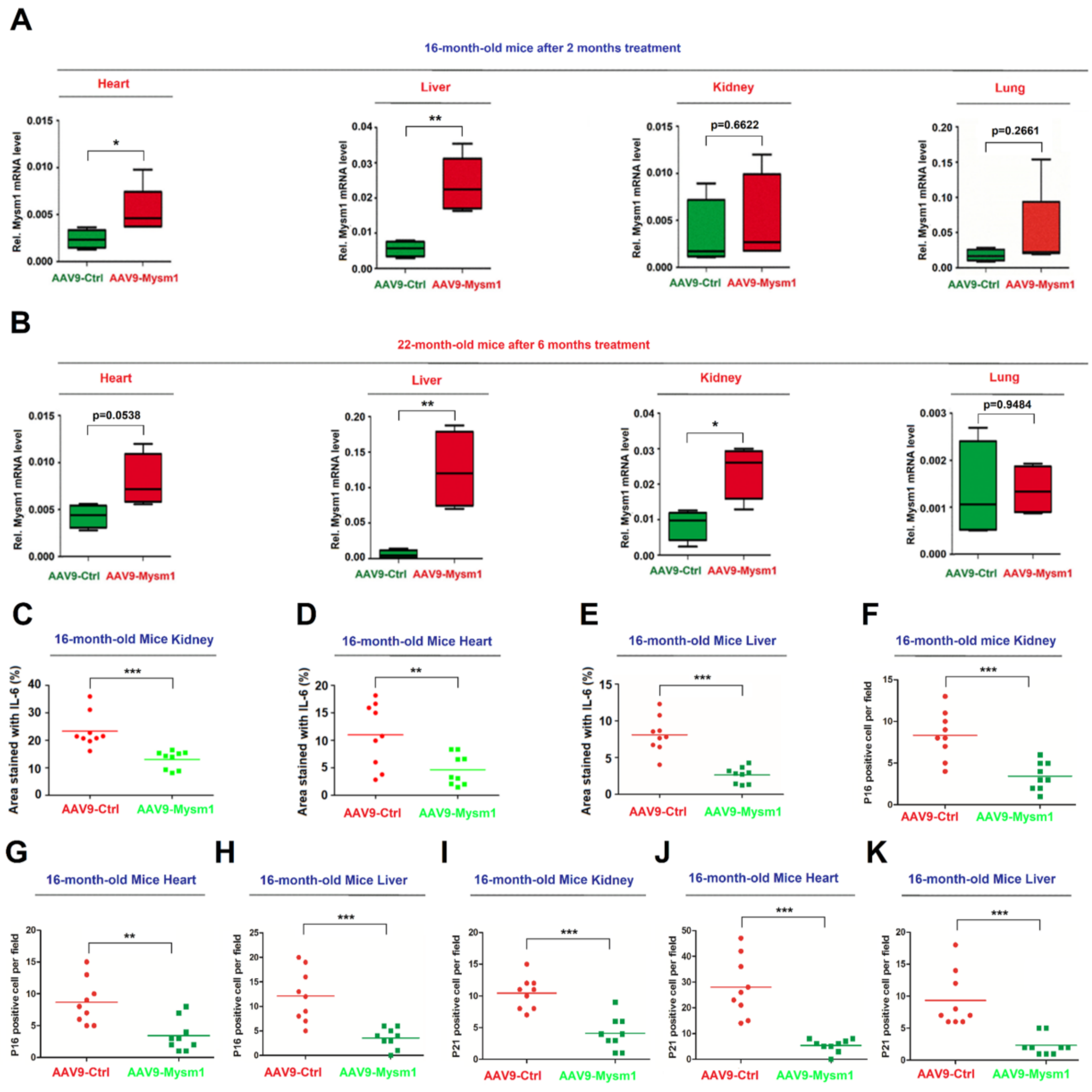

Figure S8. MYSM1 represses cytokine prodction in mice. (**A** and **B**) 14-month old WT mice were received an intraperitoneal injection (2.5 x 10^{11} MOI) of AAV9-Ctrl (n = 4) or AAV9-Mysm1 (n = 5) for 2 months (A). 16-month old WT mice were were received an intraperitoneal injection (2.5 x 10^{11} MOI) with AAV9-Ctrl (n = 5) or AAV9-Mysm1 (n = 5) for 6 months (B). The levels of of Mysm1 expressed in the heart, liver, kidney, and lung of the treated mice were quantified by RT-PCR. (**C**–**K**) 14-month old WT mice were received an intraperitoneal injection (2.5 x 10^{11} MOI) of AAV9-Ctrl (n = 4) or AAV9-Mysm1 (n = 5) for 2 months. Statistical analysis of IHC images for IL-6 protein (C–

E), p16 positive cells per field (F–H), and p21 positive cells per field (I–K) in the kidney (C, F, and I), heart (D, G, and J), and liver (E, H, and K) of the treated mice. 3–5 fields of view were selected in a double-blinded manner and analyzed using Image J. Data are means ± SD. Statistical analyses were done by using Prism software by Unpaired t-tests. $*p < 0.05$, $**p < 0.01$, $***p < 0.001$, n.s. = no significance; n = 4 for each sample group (A, B). n = 3 for each sample group (C–K).

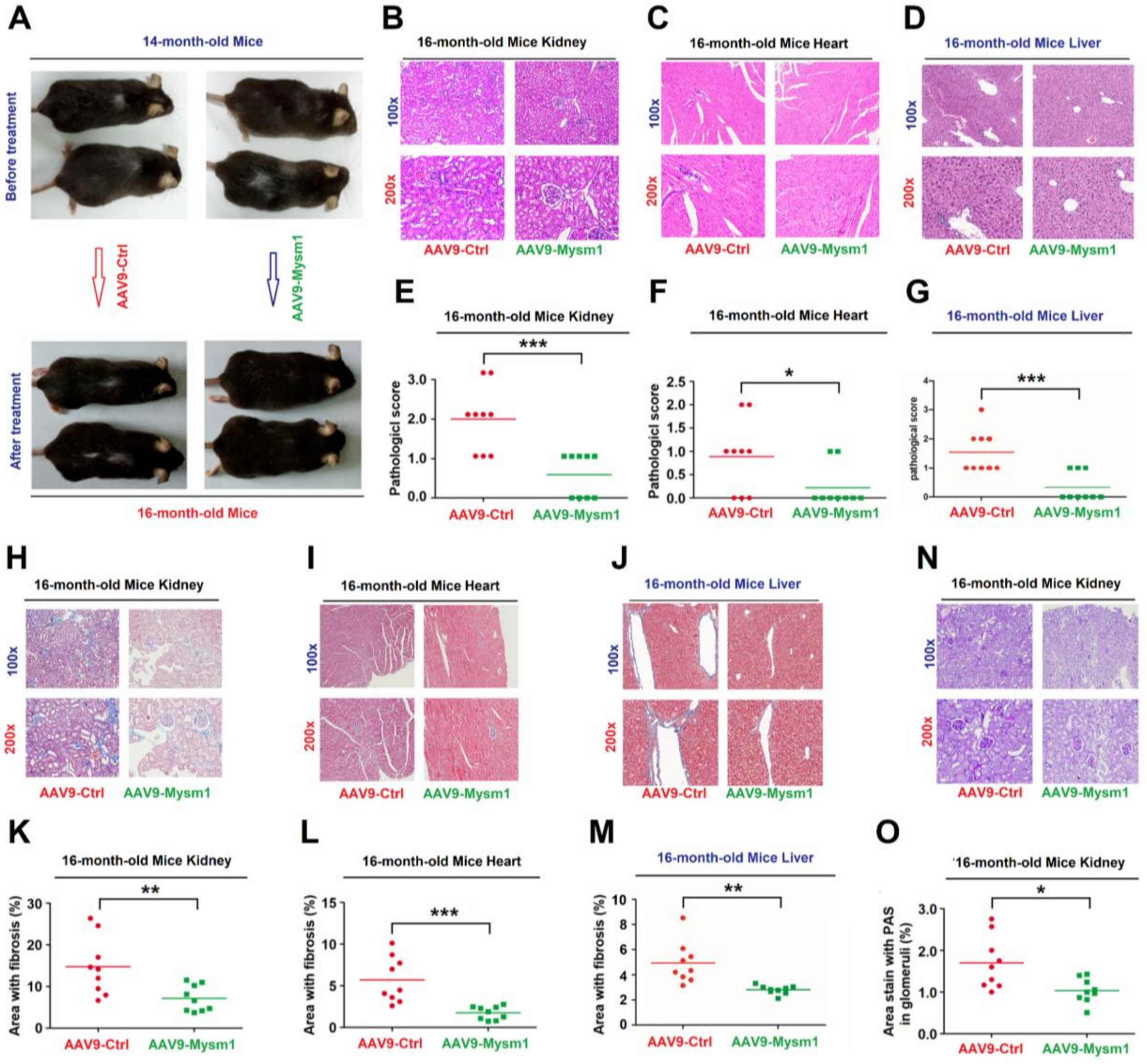

Figure S9. MYSM1 suppresses aging process in mice. 14-month old WT mice were received an intraperitoneal injection (2.5 x 10^{11} MOI) of AAV9-Ctrl (n = 4) or AAV9-Mysm1 (n= 5) for 2 months. (**A**) Images of representing mice before and after treatment were shown. (**B**–**G**) H&E stained images of the kidney, heart, and liver of mice treated with AAV9-Ctrl or AAV9-Myms1 (n = 3 per group) (B–D). Tissue pathology scores were determined as described in the materials and methods base on 3 random fields of view. Statistical analysis of the pathological scores of the kidney, heart, and liver of mice treated with AAV9-Ctrl or AAV9-Msym1 (E). (**H**–**M**) Masson

trichrome staining analyses of kidney, heart, and liver of mice treated with AAV9-Ctrl or AAV9-Myms1 (n = 3 per group) (H–J). Statistical analysis of the sizes of fibrotic lesions in the tissues upon AAV9-Mysm1 treatment (K–M). (**N** and **O**) PAS analyses of kidney of mice treated with AAV9-Ctrl or AAV9-Myms1 (n = 3 per group). For statistical analyses of IHC images, 3–5 fields of view were selected in a double-blinded manner and analyzed using ImageJ to determine the percentage of fibrotic or PAS-stained area. Statistical analysis of the pathological scores of the kidneys, hearts, and livers of mice treated with AAV9-Ctrl or AAV9-Msym1 (n = 3). Data are means ± SD. Statistical analyses were done by using Prism software by Unpaired t-tests. *p < 0.05, **p < 0.01, ***p < 0.001, n.s. = no significance. n = 3 for each sample group.

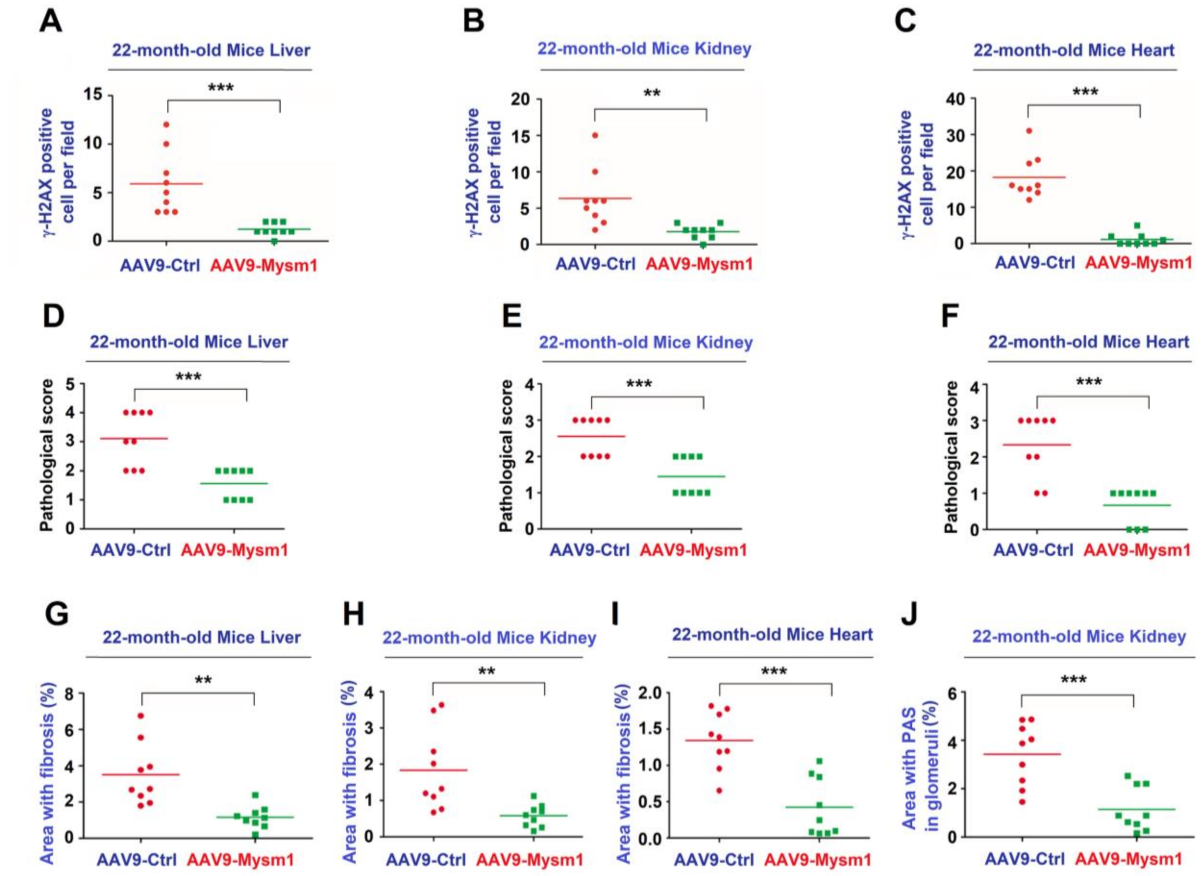

Figure S10. MYSM1 overexpression suppresses aging-related pathology. 16-month old WT mice were received an intraperitoneal injection (2.5 x 10^{11} MOI) with AAV9-Ctrl (n = 5) or AAV9-Mysm1 (n = 5) for 6 months. (**A**–**C**) Statistical analyses of the levels of γ-H2AX strain in the liver (A), kindey (B), and heart (C) of treated mice and control mice. (**D**–**F**) Statistical analyses of pathologic scores detected by IHC in the liver (D), kindey (E), and heart (F) of treated mice and control mice. (**G**–**I**) Statistical analyses of glomerular sclerosis determined by PAS staining in the liver (G), kidney (H), and heart (I) of AAV9-Mysm1-treated mice and AAV9-Ctrl-treated mice. Data are means ± SD. Statistical analyses were done by using Prism software by Unpaired t-tests. *p < 0.05, **p < 0.01, ***p < 0.001, n.s. = no significance. n = 3 for each sample group.

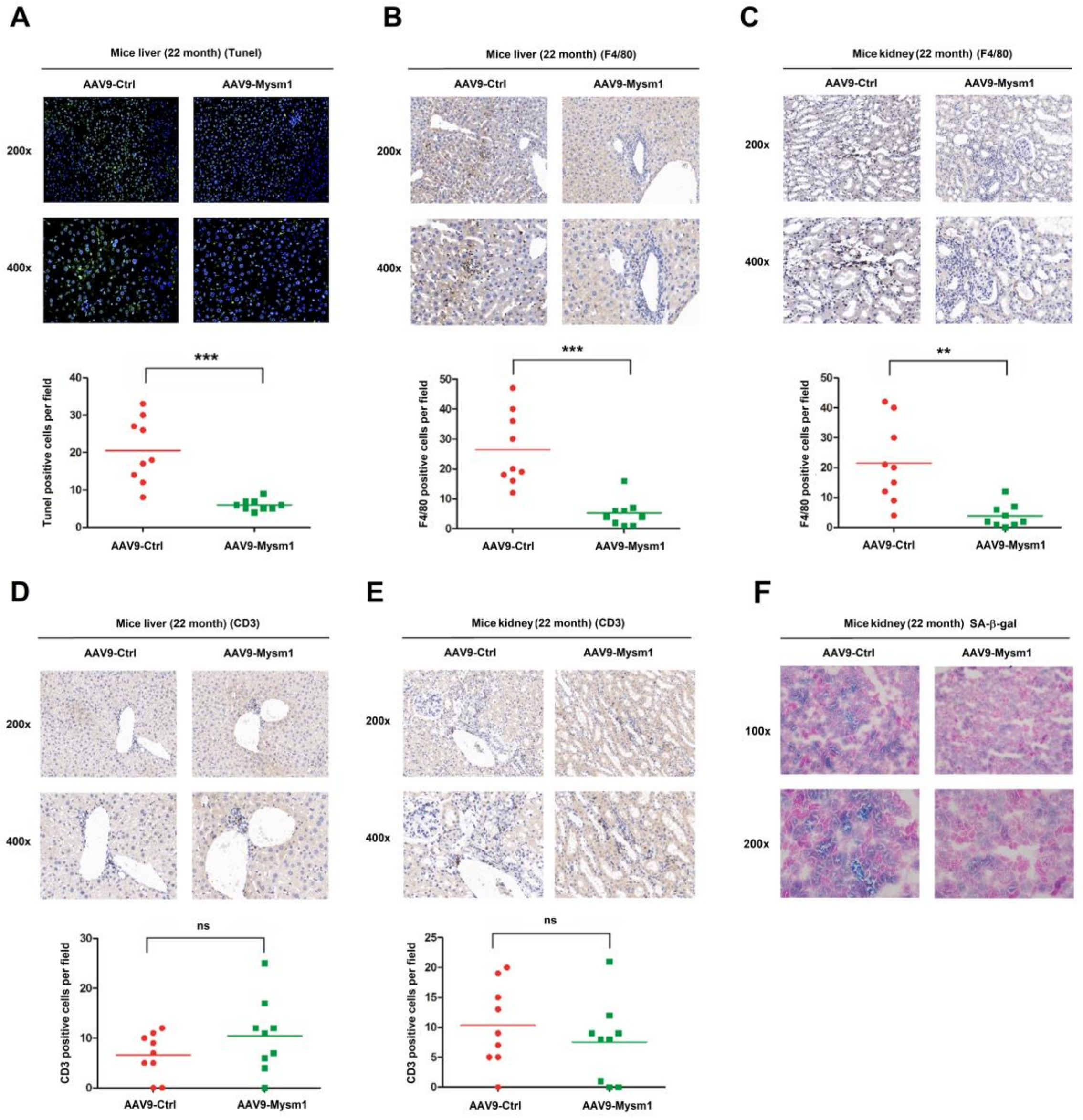

Figure S11. MYSM1 overexpression suppresses aging-related apoptosis and inflammation. (**A**–**E**) 16-month old WT mice were received an intraperitoneal injection (2.5 x 10^{11} MOI) with AAV9-Ctrl (n = 5) or AAV9-Mysm1 (n = 5) for 6 months. (**A**) TUNEL stained images of the liver of mice treated with AAV9-Ctrl or AAV9-Myms1 (n = 3 per group) (Upper). Statistical analyses of the levels of TUNEL strain in the liver (Bottom). (**B** and **C**) F4/80 stained images of the liver (B, Upper) and kidney (C, Upper) of mice treated with AAV9-Ctrl or AAV9-Myms1. Statistical analysis of the

pathological scores of the liver (B, Bottom) and kidney (C, Bottom) of mice treated with AAV9-Ctrl or AAV9-Msym1. (**D** and **E**) CD3 stained images of the liver (D, Upper) and kidney (E, Upper) of mice treated with AAV9-Ctrl or AAV9-Myms1. Statistical analysis of the pathological scores of the liver (D, Bottom) and kidney (E, Bottom) of mice treated with AAV9-Ctrl or AAV9-Msym1. (**F**) Senescence-associated β-galactosidase (SA-β-Gal) activities were determined in the kidneys of mice treated with AAV9-Ctrl or AAV9-Msym1. Data are means ± SD. Statistical analyses were done by using Prism software by Unpaired t-tests. *p < 0.05, **p < 0.01, ***p < 0.001, n.s. = no significance. n = 3 for each sample group.

Supplementary Tables

Table S1. Primers used in this study.

**Name**	**Forward**	**Reverse**
Murine IL-1α	5’-AGGAGAGCCGGGTGACAGTA-3’	5’-TCAGAATCTTCCCGTTGCTTG-3’
Murine IL-8	5’-CTGGTCCATGCTCCTGCTG-3’	5’-GGACGGACGAAGATGCCTAG-3’
Murine IL-6	5’-TCTGCAAGAGACTTCCATCCAGTTGC-3’	5’-AGCCTCCGACTTGTGAAGTGGT-3’
Murine GAPDH	5’-ACGGCCGCATCTTCTTGTGCA-3’	5’-ACGGCCAAATCCGTTCACACC-3’
Murine Mysm1	5’- AGG CAG GAC TCA AGT GGA AA-3	5’-CAG GAA TCG CTT GCT TTT CT-3’
Murine IFN-β	5’-TCCTGCTGTGCTTCTCCACCACA-3’	5’-AAGTCCGCCCTGTAGGTGAGGTT-3’.
Murine p21	5’-CTGGTGATGTCCGACCTGTT-3’	5’-TCAAAGTTCCACCGTTCTCG-3’

Murine p16	5’-GATTCAGGTGATGATGATGGGC-3’	5’-TGCACCGTAGTTGAGCAGAAG-3’
Murine MMP3	5’- TGGAGCTGATGCATAAGCCC-3’	5’- TGAAGCCACCAACATCAGGA-3’
Murine IL-1β	5’- CCAAAAGATGAAGGGCTGCT-3’	5’- TCATCAGGACAGCCCAGGTC-3’
Human p21	5’-AGAACCCATGCGGCAGCAAG-3’	5’-TGGATGCAGCCCGCCATTAG-3’
Human p16	5’-TCCCTCAGACATCCCCGATTG-3’	5’-AAATGAAAACTACGAAAGCGG-3’
Human IL-1α	5’-TGTAAGCTATGGCCCACTCCA3’	5’-AGAGACACAGATTGATCCATGCA-3’
Human IL-8	5’- ACATGACTTCCAAGCTGGCC-3’	5’- CAGAAATCAGGAAGGCTGCC-3’
Human MMP3	5’-GGATGCCAGGAAAGGTTCTG-3’	5’- CCAGGTGTGGAGTTCCTGATGT-3’
Human IL-6	5’-CAGCCCTGAGAAAGGAGACAT-3’	5’-GGTTCAGGTTGTTTTCTGCCA-3’

Human Mysm1	5’-TACAAAACCAGCCAGTTACTCAG-3’	5’ -ACTTGTAAAACAGTGCGGCTTCC-3’
Human GAPDH	5’-CGGAGTCAACGGATTTGGTC-3’	5’-GACAAGCTTCCCGTTCTCAG-3’

Supplementary Videos

Video S1. 8-month old Mysm1 wild-type (WT) C57BL/6 mice (Mysm1$^{+/+}$ C57BL/6).

Video S2. 8-month old Mysm1 knock-out (KO) C57BL/6 mice (Mysm1$^{-/-}$ C57BL/6).

Video S3. Two groups of 16-month old Mysm1 WT C57BL/6 mice before the treatments of AAV9-Ctrl and AAV9-Mysm1.

Video S4. 22-month old WT C57BL/6 mice after treated with AAV9-Ctrl for 6 months.

Video S5. 22-month old WT C57BL/6 mice after treated with AAV9-Mysm1 for 6 months.
